# RAD18 facilitates cancer progression and immunosuppression via the AKT/mTOR/c-Myc axis: a multi-omics analysis

**DOI:** 10.1038/s41698-026-01468-0

**Published:** 2026-05-11

**Authors:** Jinfeng Zhu, Qian Huang, Qingchun Liang, Wenjun Yi

**Affiliations:** 1 Department of General Surgery, The Second Xiangya Hospital of Central South University, Changsha, Hunan China; 2 Clinical Research Center For Breast Disease In Hunan Province, Changsha, Hunan China; 3 Hunan Provincial University Key Laboratory of the Fundamental and Clinical Research on Functional Nucleic Acid, the First Affiliated Hospital of Changsha Medical University, Changsha, Hunan China; 4 Department of General Practice, Shanghai East Hospital, Tongji University School of Medicine, Shanghai, China; 5 Department of Pathology, The Second Xiangya Hospital, Central South University, Changsha, Hunan China; 6 Hunan Clinical Medical Research Center for Cancer Pathogenic Genes Testing and Diagnosis, Changsha, Hunan China

**Keywords:** Biomarkers, Cancer, Computational biology and bioinformatics, Oncology

## Abstract

RAD18, a key E3 ubiquitin ligase implicated in DNA repair and genome stability, is tightly linked to cancer malignant progression, yet its pan-cancer prognostic and immunotherapeutic value remains poorly defined. Herein, we performed a comprehensive pan-cancer multi-omics analysis and validated the oncogenic mechanisms of RAD18 in liver hepatocellular carcinoma, pancreatic adenocarcinoma and triple-negative breast cancer via in vitro and in vivo assays. RAD18 was significantly overexpressed in 22 cancer types, associated with gene mutation, hypomethylation, poor prognosis and favorable diagnostic efficacy, serving as an independent poor prognostic factor for multiple cancers. Mechanistically, RAD18 promoted tumor proliferation, migration and immunosuppressive microenvironment remodeling by activating the AKT/mTOR/c-Myc axis and regulating TGF-β1/PD-L1 expression. Moreover, low RAD18 expression predicted a favorable response to immune checkpoint blockade therapy, while its high expression correlated with anti-PD-1 resistance in triple-negative breast cancer. Collectively, our findings identify RAD18 as a potential pan-cancer prognostic biomarker and immunotherapeutic target, with targeting RAD18 holding promise for reversing immunotherapy resistance in solid tumors.

## Introduction

Cancer, as a complex chronic disease, has become a major cause of death worldwide^1,2^. DNA damage tolerance (DDT), along with mutagenesis represents crucial characteristics of malignant cells, which can not only drive the occurrence of malignant tumors, but also help cancer cells resist treatment^3^. DDT encompasses a set of mechanisms enabling cell survival amidst DNA replication-associated damage^4^. In saccharomyces cerevisiae, the DDT cascade unfolds in the following manner. The RAD16 - RAD18 complex triggers the monoubiquitination of the K164 residue on proliferating cell nuclear antigen (PCNA) in response to DNA damage, which can facilitate translesion synthesis (TLS) and heighten the probability of mutagenesis. Subsequently, the MMS2 - UBC13 - RAD5 complex catalyzes the polyubiquitination of PCNA precisely at the K164 residue, which promotes an error-free mechanism for bypassing DNA lesions^4^. In higher eukaryotes, the DDT pathway involves a variety of specific DNA polymerases of the Y-family that cause error-prone TLS. The activation of this TLS polymerase usually depends on the E3 ubiquitin ligase RAD18^5^. Given the above, RAD18 promotes DNA cross-damage synthesis to prevent replication fork collapse while giving cells tolerance to DNA damage^6^, resulting in a significantly increased risk of cell carcinogenesis, making it a strong candidate for a carcinogenic factor.

Previous research has demonstrated that RAD18 exhibits a strong association with the onset and progression of specific malignant neoplasms, as well as the resistance of cancerous cells to radiotherapy and chemotherapy. As an illustration, elevated expression of RAD18 has been observed in esophageal squamous cell carcinoma (ESCC) in humans, which facilitates the migration, invasion, and in vivo colonization of cancerous cells via the JNK-MMPs signaling pathway^7^. In human gastric cancer (GC) tissues, a remarkable upregulation of the RAD18 expression level is detected compared to that in normal tissues. Silencing the RAD18 gene expression restrains the propagation and incursion of human GC cells through the inhibition of monoubiquitinated PCNA^8^. RAD18 is also involved in the partial regulation of SERTAD2 by REV1, promoting the occurrence of lung tumors^9^. In addition, several investigations have demonstrated that the up-regulated expression of RAD18 promotes radiotherapy resistance in pancreatic cancer, thyroid cancer, and glioma^10,11^. The interplay between ADAR1 and RAD18 contributes to the establishment of radioresistance in non-small cell lung cancer^12^. Two pieces of research delved into the function of RAD18 in conferring resistance to chemotherapy among tumors. One of them found that overexpressed class I HDACs regulated RAD18 expression and promoted temozolomide resistance in glioma cells^13^. An additional investigation revealed that microRNA - 379 - 5p augmented the resistance to cisplatin in ovarian cancer by upregulating the RAD18/Polη axis^14^. Nonetheless, these investigations have merely assessed the tumor-promoting function of RAD18 in a restricted and particular set of cancer types. The manifestation of RAD18 across diverse cancers and the fundamental mechanisms propelling cancer progression still remain ambiguous.

Pan-cancer analysis represents an all-encompassing methodology for the investigation of a diverse spectrum of cancer types. It can identify common oncogenic drivers that operate in different cancer lineages and promote the development of new therapeutic targets^15^. This approach changes the traditional model of treating tumors based on their origin or location, and focuses more on specific genetic abnormalities that cause malignant tumor growth^16^. Moreover, immune checkpoint blockade (ICB) has also impacted traditional cancer treatment methods and has produced lasting survival benefits for some cancer patients. Nevertheless, a large number of patients still do not benefit from it, suggesting that it is necessary to fully consider whether it can well predict immune response when identifying a new target^17^. In general, pan-cancer analysis helps us to have a deeper understanding of the occurrence and development mechanism of cancer from multiple dimensions such as transcriptome, epigenome and tumor immunity^18,19^. As far as we know, this is the first pan-cancer study on the carcinogenic effect of RAD18, which specifically covers the RAD18 gene expression pattern, correlation with clinical indicators, genetic changes, changes in epigenetic modifications, value in prognostic assessment and diagnosis, tumor immune microenvironment landscape, body immune response status, prediction of small molecule drug sensitivity, in vitro experimental verification, etc. This provides initial key clues for exploring and optimizing the application of RAD18 targeting strategies in a wider range of cancer types in the future. The full names and abbreviations of all cancer types can be found in Supplementary Table 1.

## Results

### Expression, subcellular localization and clinical characteristics of RAD18 across the spectrum of cancers

**Fig. 1:**
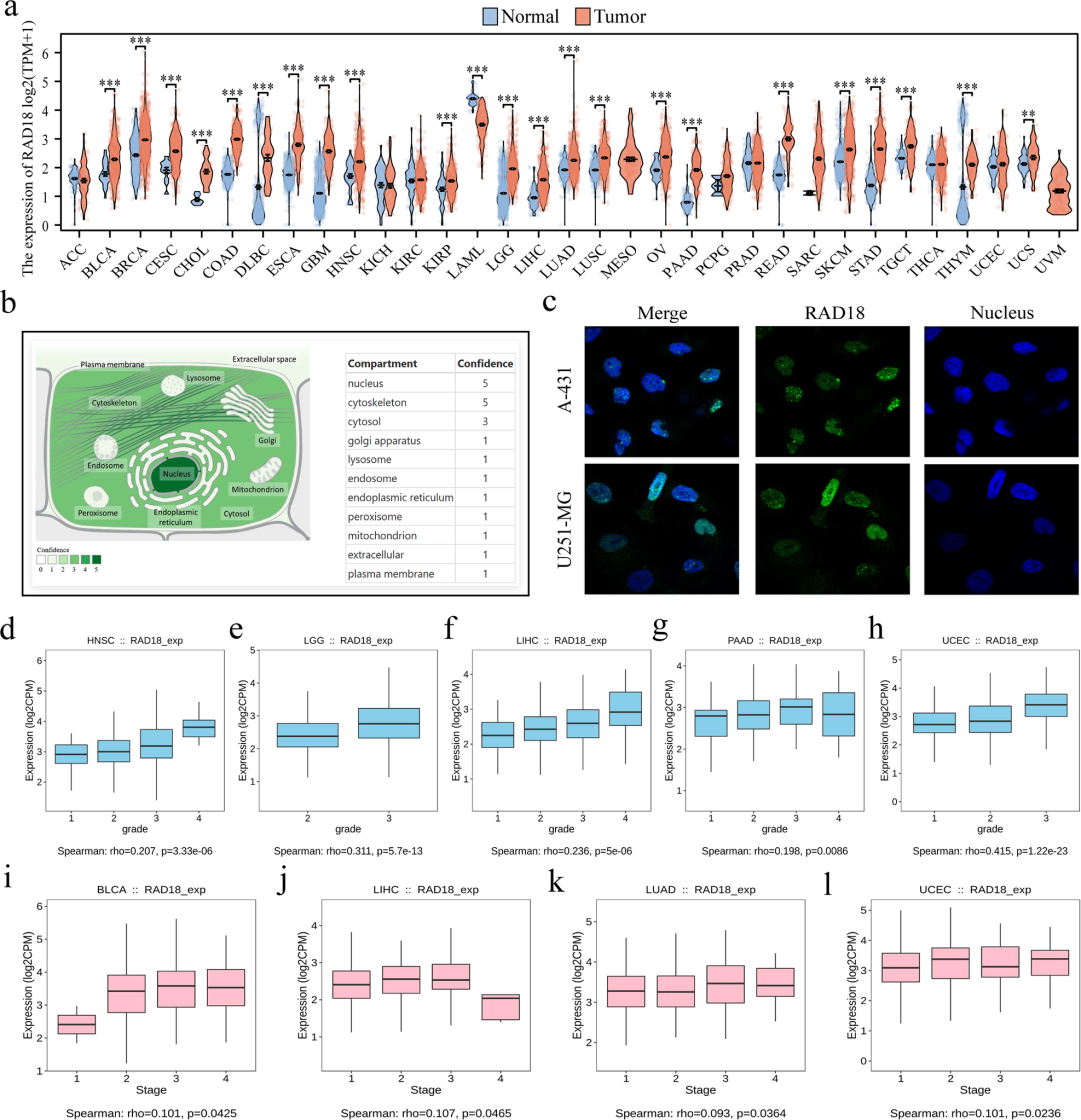
Expression pattern, localization, and clinical characteristics of RAD18 in pan-cancer. **a** The difference in RAD18 expression between cancer tissues and corresponding normal tissues in 33 cancer types. **b, c** The subcellular localization map (**b**) and immunofluorescence map (**c**) of RAD18 were derived from the Genecards database and Human Protein Atlas. **d**–**h** The association between RAD18 mRNA expression and pathological grade was evaluated by Spearman’s correlation analysis in HNSC (**d**), LGG (**e**), LIHC (**f**), PAAD (**g**), and UCEC (**h**). **i**–**l** The association between RAD18 mRNA expression and the pathological stage was evaluated by Spearman’s correlation analysis in BLCA (**i**), LIHC (**j**), LUAD (**k**), and UCEC (**l**). **p < 0.01; ***p < 0.001; and unmarked asterisks indicate no statistical significance between the two groups.

Leveraging the TCGA + GTEx cohort, we discerned that the expression level of RAD18 mRNA was significantly upregulated in 22 distinct tumor tissues when juxtaposed with their corresponding normal tissues. These tumor types included BLCA, BRCA, CESC, CHOL, COAD, DLBC, ESCA, GBM, HNSC, KIRP, LGG, LIHC, LUAD, LUSC, OV, PAAD, READ, SKCM, STAD, TGCT, THYM, and UCS (all *p* < 0.01, Fig. 1a). Notably, RAD18 mRNA expression was exclusively downregulated in LAML tissues (Fig. 1a). Moreover, no significant disparity in the expression of RAD18 mRNA was detected between tissues of ACC, KICH, KIRC, PCPG, PRAD, SARC, THCA, UCEC and their respective normal counterparts (Fig. 1a). Analysis of the HPA database revealed tissue-specific RAD18 protein expression in normal tissues, with high expression in the thyroid, colorectum, and bladder, moderate expression in the esophagus, stomach, and kidney, low expression in the pancreas, prostate, and ovary, and no expression in the lung, liver, or breast (Supplementary Fig. 1a). In tumor tissues, the nuclear positivity rate varied significantly across cancer types, with high rates in head and neck tumors, testicular cancer, and urothelial carcinoma, and lower rates in thyroid cancer, carcinoid tumors, and prostate cancer (Supplementary Fig. 1b). Immunohistochemical comparison revealed weak protein staining in normal tissues (endometrium, cerebral cortex, pancreas, and liver), while staining intensity increased significantly in tumor tissues (endometrial cancer, glioma, pancreatic cancer, and liver cancer), suggesting that RAD18 expression and localization vary during tumor development and progression (Supplementary Fig. 1c).

RAD18 predominantly localizes to the nucleus and cytoskeleton of cells (Fig. 1b). To intuitively present the subcellular expression of RAD18 protein, we retrieved immunofluorescent images of RAD18 in A-431 and U251-MG cells from the HPA database (Fig. 1c). Analysis via the TISIDB database revealed an association between RAD18 expression and the pathological grade and stage of the tumor. Specifically, with the increase in grade, RAD18 mRNA expression was significantly upregulated in HNSC, LGG, LIHC, PAAD, and UCEC (Fig. 1d–h). In BLCA, LIHC, LUAD, and UCEC, RAD18 mRNA expression was also significantly upregulated with the increase in stage level (Fig. 1i–l).

### Potential determinants of abnormal expression of RAD18 across the spectrum of cancers

**Fig. 2:**
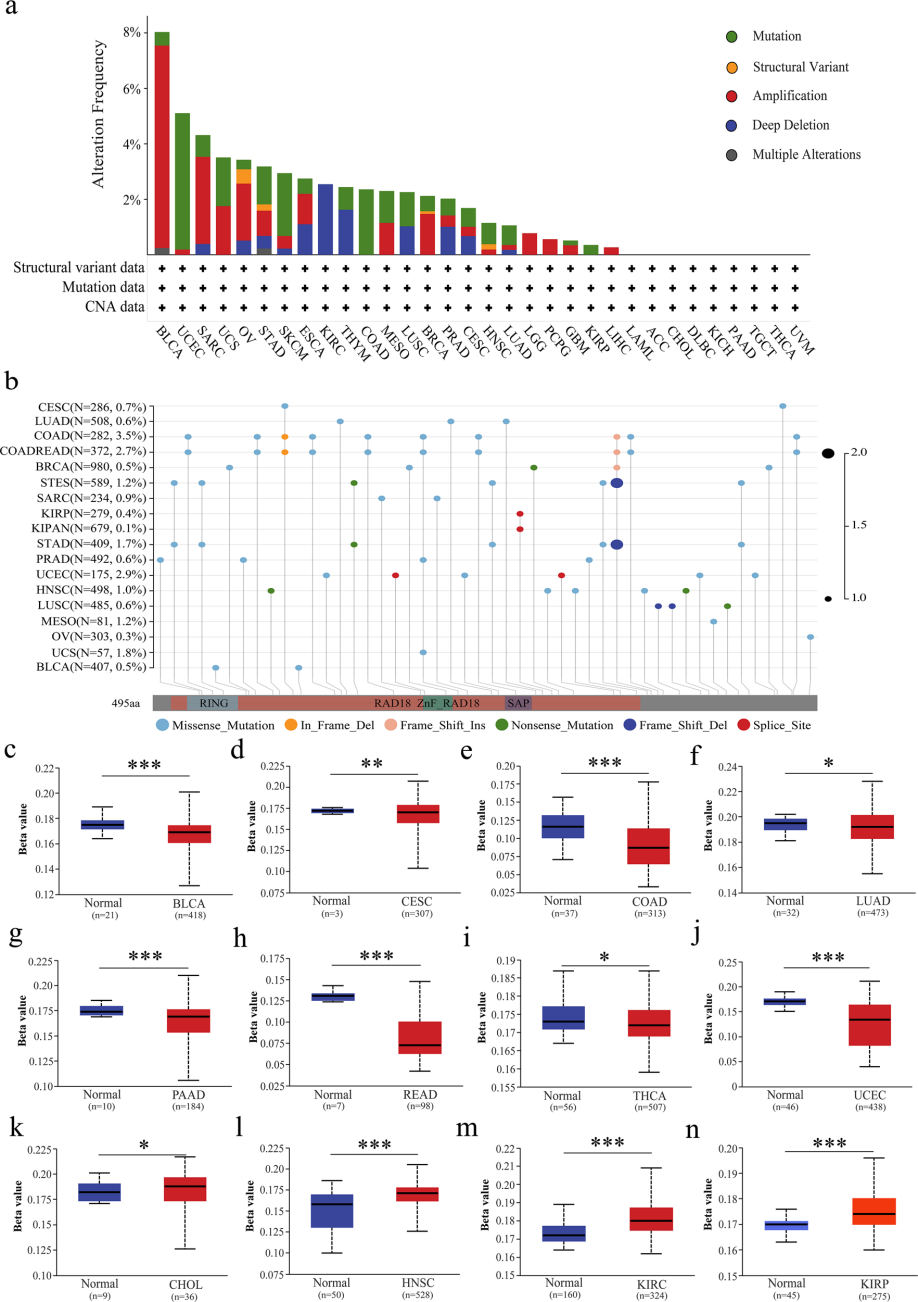
Potential influencing factors of abnormal expression of RAD18 in pan-cancer. **a** Genetic alteration of RAD18 in 32 cancer types. **b** Gene alteration types, sites, and number of cases of RAD18 in samples of various cancer types. **c**–**j** RAD18 DNA promoter methylation levels were lower in BLCA (**c**), CESC (**d**), COAD (**e**), LUAD (**f**), PAAD (**g**), READ (**h**), THCA (**i**), and UCEC (**j**) cancer tissues than in the corresponding normal tissues. **k**–**n** RAD18 DNA promoter methylation levels were higher in CHOL (**k**), HNSC (**l**), KIRC (**m**), and KIRP (**n**) cancer tissues than in the corresponding normal tissues. COADREAD, colorectal adenocarcinoma; KIPAN, the kidney pan-cancer. *p < 0.05; **p < 0.01; and ***p < 0.001.

To explore the potential determinants of aberrant RAD18 expression across diverse cancer types, we assessed the genetic variation profiles and epigenetic alterations of RAD18. The genetic variation results showed that among the 32 tumor tissues, RAD18 presented five genetic changes mainly characterized by amplification, mutation, and deep deletion in most tumors (about 72%) (Fig. 2a). Among them, “amplification” mainly occurred in BLCA (7.3%), SARC (3.14%), OV (2.05%), BRCA (1.48%), LGG (0.78%), PCPG (0.56%), GBM (0.34%), and LIHC (0.27%). “Mutation” was mainly found in UCEC (4.9%), SKCM (2.26%), COAD (2.36%), LUSC (1.23%), HNSC (0.76%), LUAD (0.71%), and KIRP (0.35%). “Deep deletion” is most typical for KIRC (2.54%), THYM (1.63%), and PRAD (1.01%) (Supplementary Table 2). To explore the overall distribution characteristics of different genetic alteration types in 32 cancer types, this study calculated the average number of occurrences and proportions of each genetic alteration. The results in Supplementary Table 2 show that across all cancer types, “mutation” is the genetic alteration type with the highest average number of occurrences (2.91 times) and the highest average proportion (0.85%) in RAD18. The next most common genetic alteration types are “amplification” and “deep deletion,” with average occurrences of 2.81 and 1.16, respectively, and average proportions of 0.82% and 0.34%, respectively. However, regardless of the context, the mutation frequency of the RAD18 gene in “structural variation” and “multiple variation” is generally low. Figure. 2b additionally depicts the RAD18 genetic alteration profiles (types and loci), total specimen count, and alteration prevalence across diverse cancer specimens. We observed that missense mutations occurred at a high frequency in multiple tumors.

Reported studies indicate that DNA methylation, a crucial epigenetic modification, regulates gene expression via silencing transposable elements and mediating transcriptional repression^20^. It predominantly targets CpG islands within gene regulatory elements like promoters and enhancers^21^. Therefore, this study evaluated the DNA promoter methylation status of RAD18 across the entire spectrum of cancers. Among BLCA, CESC, COAD, LUAD, PAAD, READ, THCA, and UCEC cohorts, the DNA promoter methylation of RAD18 was conspicuously less compared with normal tissue counterparts (Fig. 2c–j), while the opposite trend was depicted in CHOL, HNSC, KIRC, and KIRP (Fig. 2k–n). We further examined the relationship between RAD18 expression and DNA promoter methylation in cancers exhibiting low RAD18 DNA promoter methylation levels. We found a significant negative correlation between RAD18 mRNA expression and methylation levels in all seven cancer cohorts, with the exception of THCA (Supplementary Fig. 2), suggesting that hypomethylation may drive RAD18 overexpression in these cancers.

### Clinical prognostic significance of RAD18 in pan-cancer

**Fig. 3:**
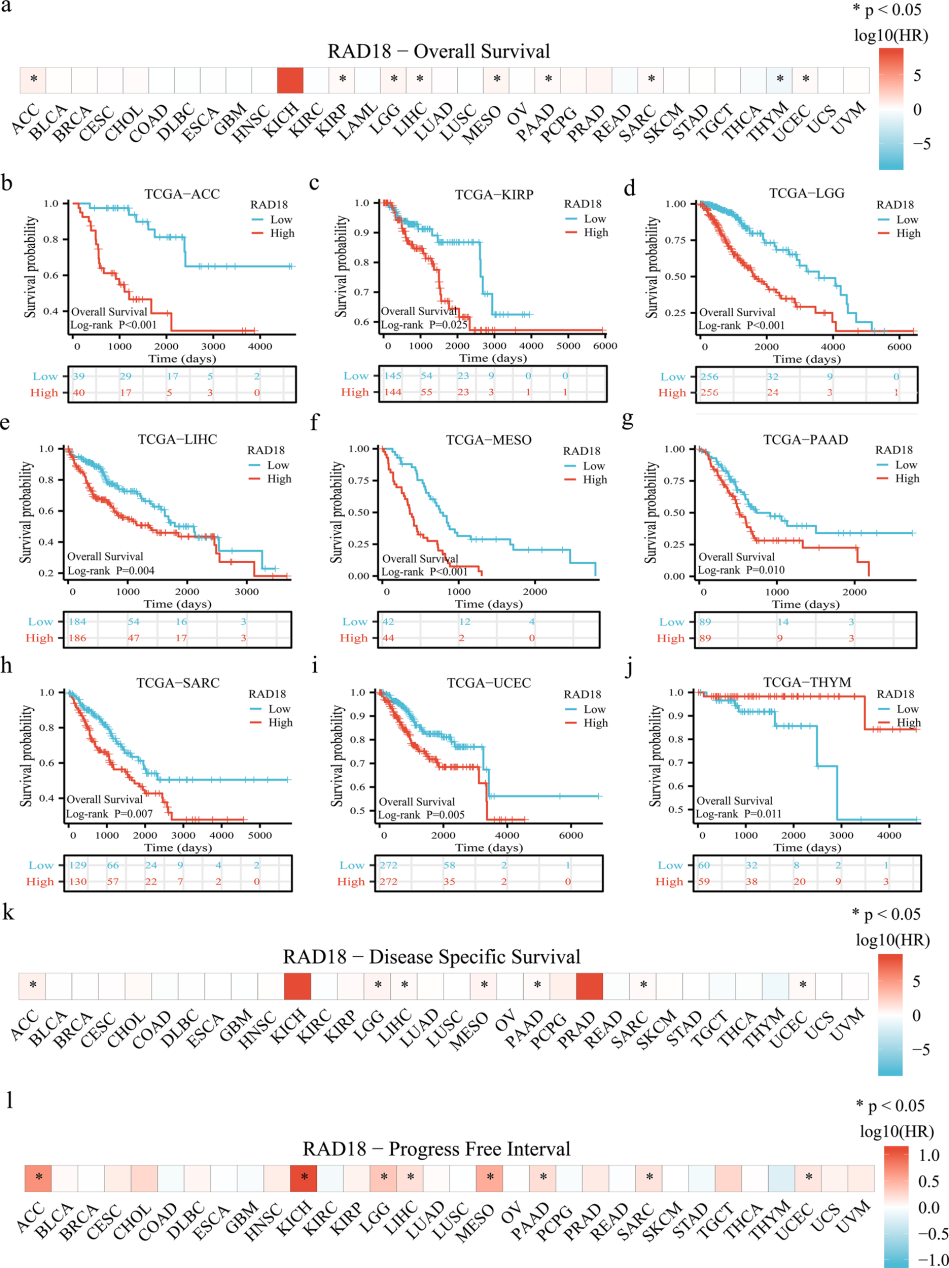
Prognostic relevance of RAD18 in pan-cancer. **a** Univariate Cox regression analysis evaluated the relationship between RAD18 expression and overall survival (OS) of cancer patients. **b**–**j** The difference in OS between RAD18 high-expresser and low-expresser subgroups in ACC (**b**), KIRP (**c**), LGG (**d**), LIHC (**e**), MESO (**f**), PAAD (**g**), SARC (**h**), UCEC (**i**) and THYM (**j**). **k, l** Univariate Cox regression analysis evaluated the relationship between RAD18 expression and disease-specific survival (**k**) and progression-free interval (**l**) of cancer patients. *p < 0.05.

According to the prognostic data provided by Liu et al.^22^, we evaluated the effect of RAD18 expression on OS, DSS and PFI of different tumors. The prognostic heat map showed that RAD18 was a potential risk factor for OS in patients with ACC, KIRP, LGG, LIHC, mesothelioma (MESO), PAAD, SARC, and UCEC, but a potential protective factor for OS in patients with THYM (Fig. 3a). By drawing the Kaplan-Meier curve and performing log-rank analysis, we observed that compared with the RAD18 low-expresser subgroup, the RAD18 high-expresser subgroup generally showed worse OS in the ACC, KIRP, LGG, LIHC, MESO, PAAD, SARC, and UCEC cohorts, and better OS in THYM (Fig. 3b–j). Considering the significant heterogeneity among different BRCA subtypes, we evaluated the expression and prognostic value of RAD18 in different BRCA subtypes. We found that RAD18 was significantly elevated in basal-like BRCA subtypes compared to normal tissue (Supplementary Fig. 3a), and that high RAD18 expression was associated with poor prognosis in both overall BRCA and basal-like BRCA (Supplementary Fig. 3b–f). RAD18 is also a potential risk factor for DSS and PFI in ACC, LGG, LIHC, MESO, PAAD, SARC, and UCEC (Fig. 3k, l). To further validate RAD18’s independent prognostic value across cancers, we performed stricter uni - and multivariate Cox regression analyses. The study found that after fully controlling a series of important clinical characteristics that are critical to tumor prognosis, such as age, gender, pathological grade, and stage, RAD18 was determined to independently predict unfavorable prognosis in ACC, MESO, LGG, LIHC and PAAD (all hazard ratios (HRs) > 1, *p* < 0.05) (Supplementary Tables 3–7). Specifically, in the aforementioned cohort, the HRs for shorter OS in the RAD18 high-expresser subgroup compared to the RAD18 low-expresser subgroup were 4.011, 2.568, 1.885, 1.726, and 1.599, respectively. This suggests that RAD18 expression levels may serve as an effective indicator for stratified management of these cancer patients. In summary, RAD18 is an effective and reliable prognostic marker, and higher RAD18 expression often indicates worse survival outcomes.

### Diagnostic worth of RAD18 across cancers

**Fig. 4:**
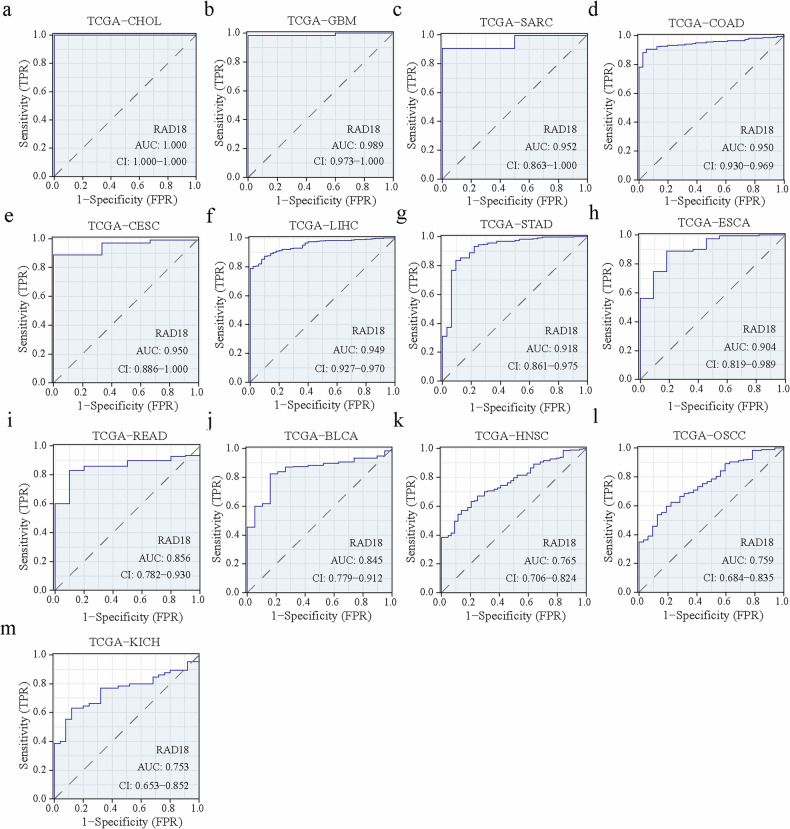
Diagnostic value of RAD18 in pan-cancer. **a**–**h** The area under the receiver operating characteristic (ROC) curve (AUC) values of RAD18 in diagnosing CHOL (**a**), GBM (**b**), SARC (**c**), COAD (**d**), CESC (**e**), LIHC (**f**), STAD (**g**), and ESCA (**h**) were all greater than 0.9. **i**–**m** The AUC values of RAD18 in diagnosing READ (**i**), BLCA (**j**), HNSC (**k**), OSCC (**l**), and KICH (**m**) were all greater than 0.75 and less than 0.9.

Given the differential expression pattern of RAD18 across diverse cancers, we also tested its potential diagnostic value by drawing ROC curves and calculating AUC values. As shown in Fig. 4, RAD18 showed excellent diagnostic performance in CHOL, GBM, SARC, COAD, CESC, LIHC, STAD, and ESCA (all AUC values > 0.90, Fig. 4a–h). Furthermore, compared with traditional diagnostic markers, RAD18 still showed significant advantages in the diagnosis of these cancer types (Supplementary Fig. 4). It also had ideal diagnostic performance in READ, BLCA, HNSC, OSCC, and KICH (all AUC values > 0.75, Fig. 4i–m). However, the diagnostic performance of RAD18 in LUSC, SKCM, KIRP, PCPG, LUAD, UCEC, THCA, KIRC, THYM, PAAD, BRCA, and PRAD was relatively poor (Supplementary Fig. 5).

### Expression and functional analysis of RAD18 in single-cell and spatial transcriptome sequencing data

**Fig. 5:**
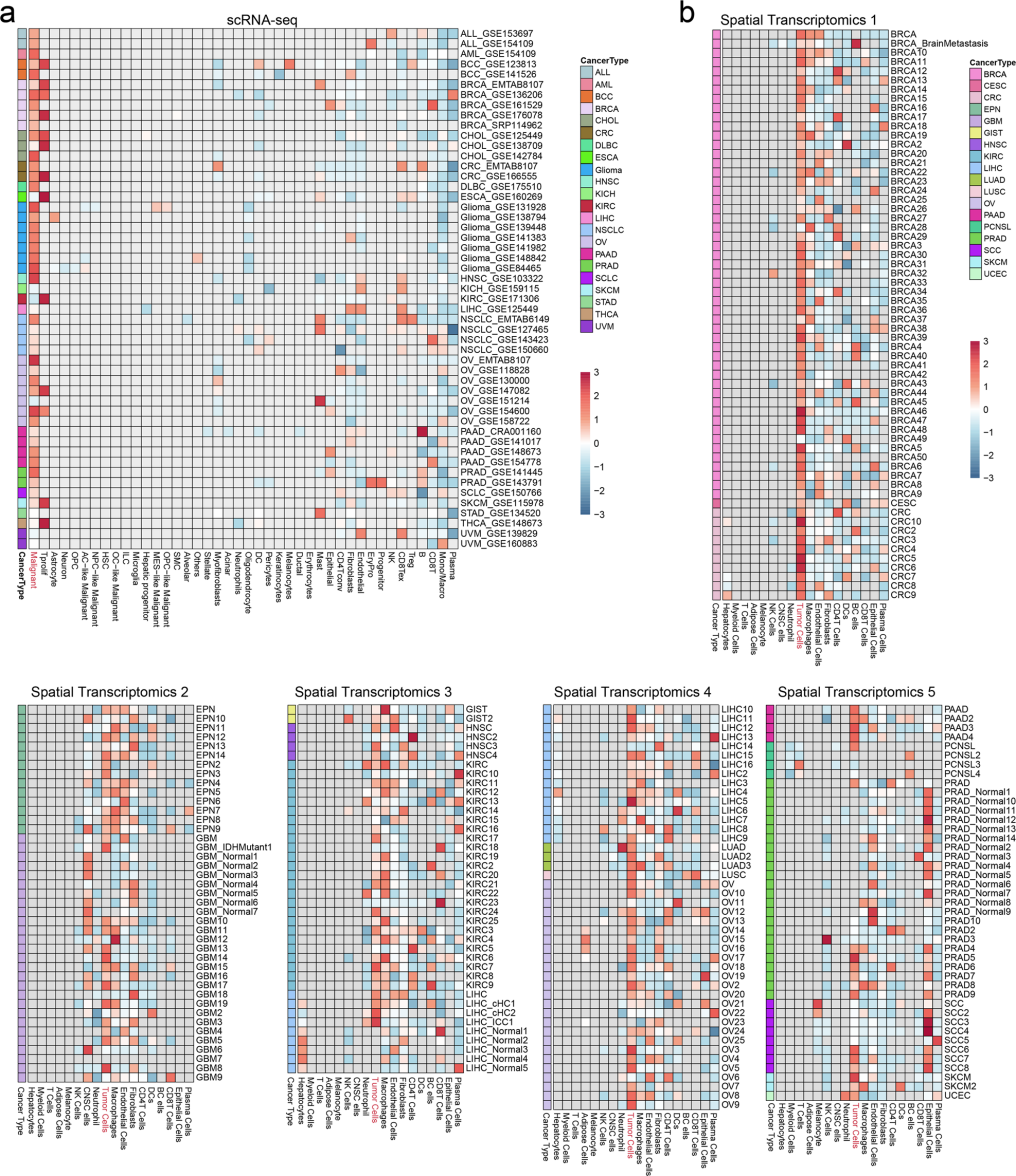
Expression of RAD18 in single-cell RNA sequencing (scRNA-seq) and spatial transcriptomics across pan-cancers. **a** Expression of the RAD18 gene detected by scRNA-seq in pan-cancers. The gradient color scale on the right maps the normalized data range: the warm color gradient (red-orange) indicates high expression levels, the cool color gradient (blue-cyan) corresponds to low expression intensities, and neutral gray denotes missing data. **b** Expression heatmap of RAD18 in microregions of different cell types within pan-cancer spatial transcriptomics sections.

Single-cell RNA sequencing (scRNA-seq) enables the dissection of gene expression profiles at single-cell resolution, serving as a powerful tool to investigate cellular heterogeneity within the tumor microenvironment (TME). By leveraging scRNA-seq data, specific gene expression patterns of distinct cell subsets can be uncovered, thereby elucidating the complexity of the TME. As shown in Fig. 5a, quantitative analysis revealed significant tissue- and cell-type-specific heterogeneity in RAD18 expression across pan-cancers: RAD18 was specifically highly expressed in malignant cell subsets, while low expression was detected in plasma cells, CD8^+^T cells and monocyte/macrophage subsets.

Deconvolution of spatial transcriptomics data based on scRNA-seq is an integrated analysis approach that combines high-resolution information from scRNA-seq with spatial transcriptomics data. Its core purpose is to delineate the distribution and composition of cell types in spatial transcriptomics data. By integrating these two datasets, cell type-specific gene expression profiles can be reconstructed at the sub-spot level, providing more detailed insights into cell type distribution and gene expression. From a pan-cancer perspective, RAD18 exhibited a marked expression advantage in malignant cell-enriched microregions, which validated the accuracy of the deconvolution results (Fig. 5b).

**Fig. 6:**
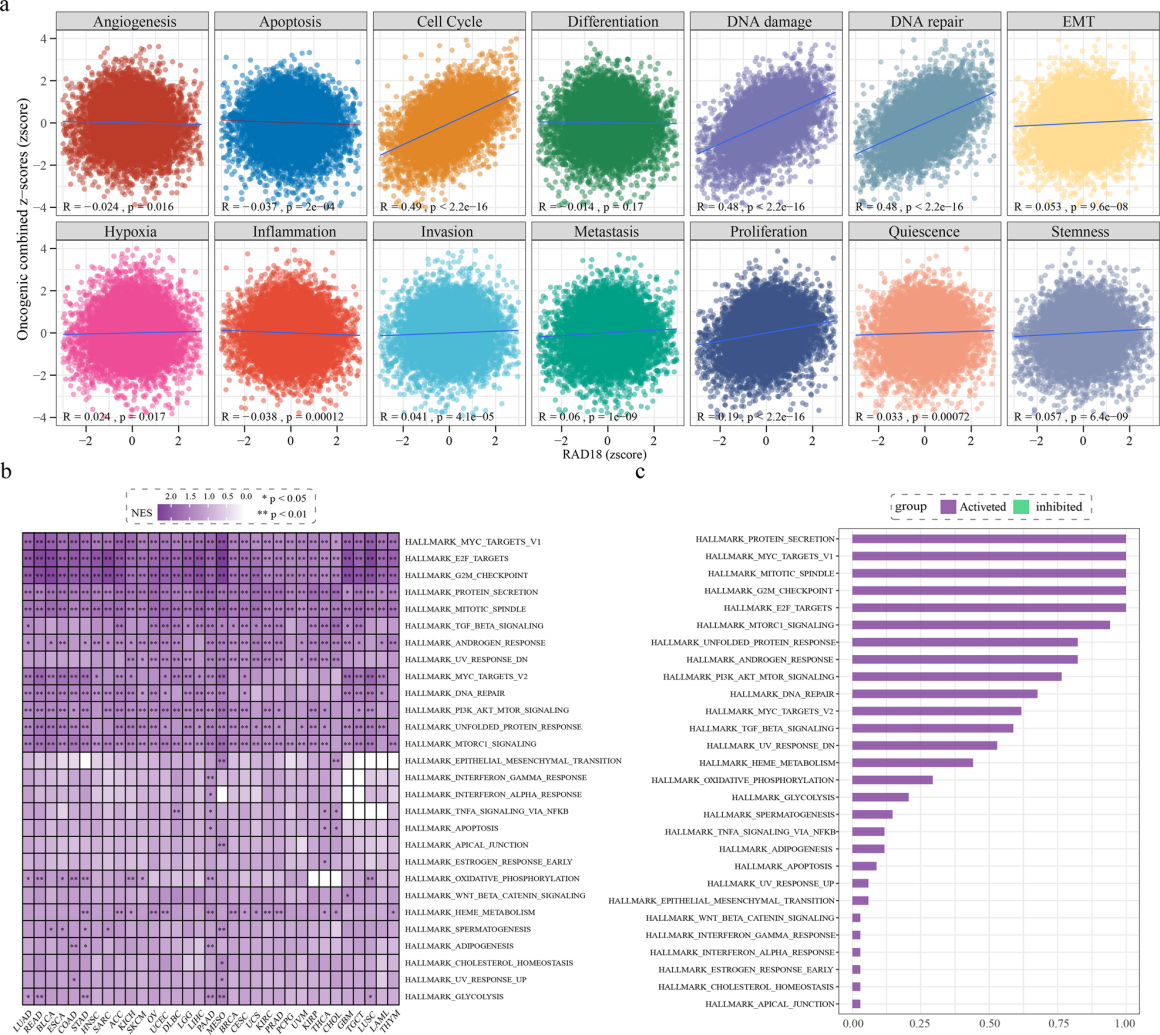
Analysis of the biological function of RAD18 in pan-cancer. **a** Correlation between RAD18 expression and 14 functional states of cancer single cells (including stemness, invasion, metastasis, proliferation, epithelial-mesenchymal transition (EMT), angiogenesis, apoptosis, cell cycle, differentiation, DNA damage, DNA repair, hypoxia, inflammation, and quiescence). The ordinate represents the *z*-score normalized values of the combined z-scores for each functional state, and the abscissa denotes the *z*-score of gene expression levels. Different colors indicate different types of functional states, and R represents the Pearson correlation coefficient. **b** Enrichment analysis results of RAD18 in the Hallmark gene set. **c** Absolute enrichment rate of RAD18 in each Hallmark gene set. NES, normalized enrichment score.

Furthermore, functional annotation via the CancerSEA database demonstrated that RAD18 expression was significantly positively correlated with cell cycle, DNA damage, DNA repair, epithelial-mesenchymal transition (EMT), hypoxia, invasion, metastasis, quiescence, and stemness. In contrast, it showed a significant negative correlation with angiogenesis, apoptosis, and inflammation (Fig. 6a).

### Biological functions of RAD18 in pan-cancer

To decipher RAD18’s latent biological functions across various cancer types, we conducted GSEA on RAD18 in 33 cancer cohorts. The results of GSEA showed that, among the Hallmark gene sets, RAD18 was significantly enriched in pathways related to cell cycle regulation and proliferation (MYC targets V1, E2F targets, G2M checkpoint, and mitotic spindle), tumor promotion (mTORC1 signaling, PI3K/AKT/mTOR signaling, TGFβ signaling, and Wnt β-catenin signaling), as well as cell metabolism, stress, and repair responses (protein secretion, oxidative phosphorylation, glycolysis, heme metabolism, unfolded protein response, and DNA repair) in most tumor types (Fig. 6b, c). This provides an important theoretical basis for the subsequent in - depth exploration of the specific mechanism of action of RAD18 in cancer.

### Immune landscape of RAD18 in pan-cancer

**Fig. 7:**
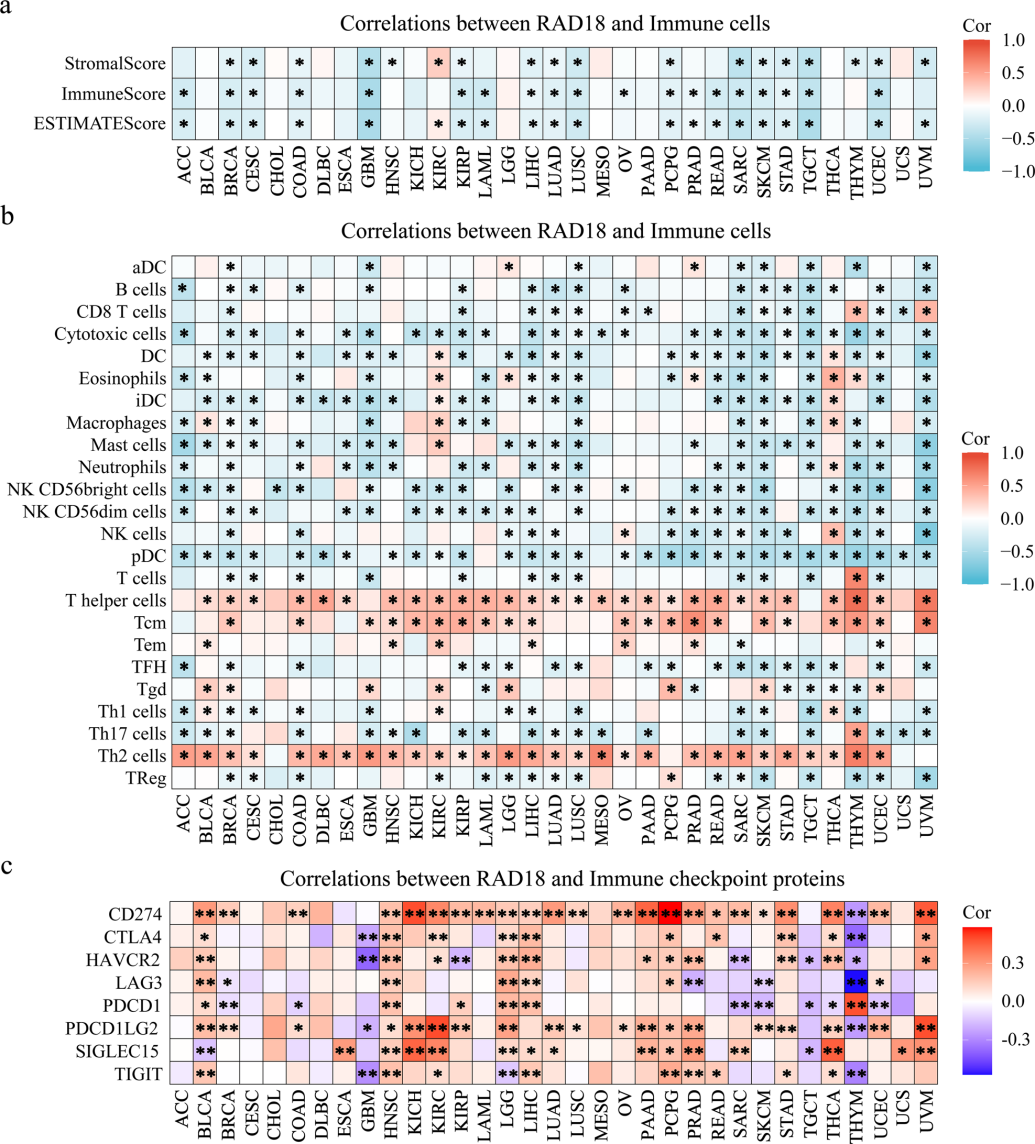
Immune landscape of RAD18 in 33 cancer types. **a** The correlation between RAD18 expression and StromalScore, ImmuneScore, and ESTIMATEScore was evaluated by the ESTIMATE algorithm. **b** The correlation between RAD18 expression and the abundance of immune cell infiltration was evaluated by the ssGSEA algorithm. **c** The relationship between RAD18 expression and immune checkpoint protein expression was evaluated by Spearman’s correlation analysis. *p < 0.05; and **p < 0.01.

Studies have shown that most oncogenic driver mutations constitutively activate signaling pathways that promote cancer cell survival and proliferation. These intrinsic oncogenic pathways can affect local and systemic immune landscapes, causing tumors to escape immune surveillance and inhibit anti-tumor immune responses^23^. Based on this background, it is particularly important to evaluate the immune landscape of RAD18 in different cancers. In the pan-cancer cohort, we observed that except for KIRC, the expression level of RAD18 showed a significant negative correlation with the stromal score, immune score, and ESTIMATE score of most cancer types (Fig. 7a). Next, we found that in most tumor cohorts, the expression of RAD18 showed a significant negative correlation with the infiltration abundance of immune cells with anti-tumor effects, such as B cells, CD8 T cells, Cytotoxic cells, NK CD56bright cells, NK CD56dim cells, NK cells, and Th1 cells (Fig. 7b). Conversely, RAD18 expression exhibited a substantial positive association with the infiltration level of immune cells predominantly having pro-tumor effects, like Th2 cells (Fig. 7b). Among the BRCA, KIRP, LIHC, LUAD, LUSC, OV, PAAD, SARC, SKCM, STAD, TGCT, and UCEC cohorts, RAD18 expression was significantly negatively correlated with CD8⁺T cell infiltration and significantly positively correlated with Th2 cell infiltration. To further accurately evaluate the relationship between RAD18 expression and these two immune cell infiltrations, we divided the above cancer cohorts into high and low immune score groups based on the median immune score and performed stratified analysis. The results of the stratified analysis showed that in the BRCA, SARC, STAD, and TGCT cohorts, regardless of whether they were in the high or low immune score group, RAD18 expression was significantly negatively correlated with CD8⁺T cell infiltration and significantly positively correlated with Th2 cell infiltration (all p < 0.05, Supplementary Fig. 6a-p). A significant positive correlation between RAD18 expression and Th2 cell infiltration was observed in both immune score groups across the LIHC, LUAD, LUSC, PAAD, SKCM, and UCEC cohorts (all *p* < 0.01), but a significant negative correlation between RAD18 expression and CD8⁺T cell infiltration could not be observed simultaneously in the two subgroups (Supplementary Fig. 7a–x). Furthermore, a positive correlation between RAD18 expression and Th2 cell infiltration, or a negative correlation with CD8⁺T cell infiltration, was only observed in the high immune score group in the OV and KIRP cohorts (Supplementary Fig. 8a–h). The outcomes of this research imply that the RAD18 gene is potentially intricately linked to the establishment and sustenance of the suppressive immune microenvironment, while also considering the heterogeneity of tumor immunity.

Immune checkpoint proteins (ICPs) are a class of proteins that prevent excessive inflammatory responses by regulating immune responses. Overexpression of certain inhibitory ICPs can help cancer cells destroy immune responses, thereby inhibiting anti-tumor immunity^24^. In this study, we found that except for GBM, TCGT and THYM, RAD18 showed a marked positive association with the expression of CD274, CTLA4, and PDCD1LG2 in most other cancers (Fig. 7c). In the HNSC, LIHC, and PCPG cohorts, RAD18 demonstrated a substantial positive link with the expression of nearly all ICPs (Fig. 7c). These findings suggest that RAD18 is pivotal in modulating the expression of ICPs and also hint at its potential for predicting the response to tumor immunotherapy.

### Pan-cancer immune response prediction by RAD18 and its link with small-molecule drug sensitivity

**Fig. 8:**
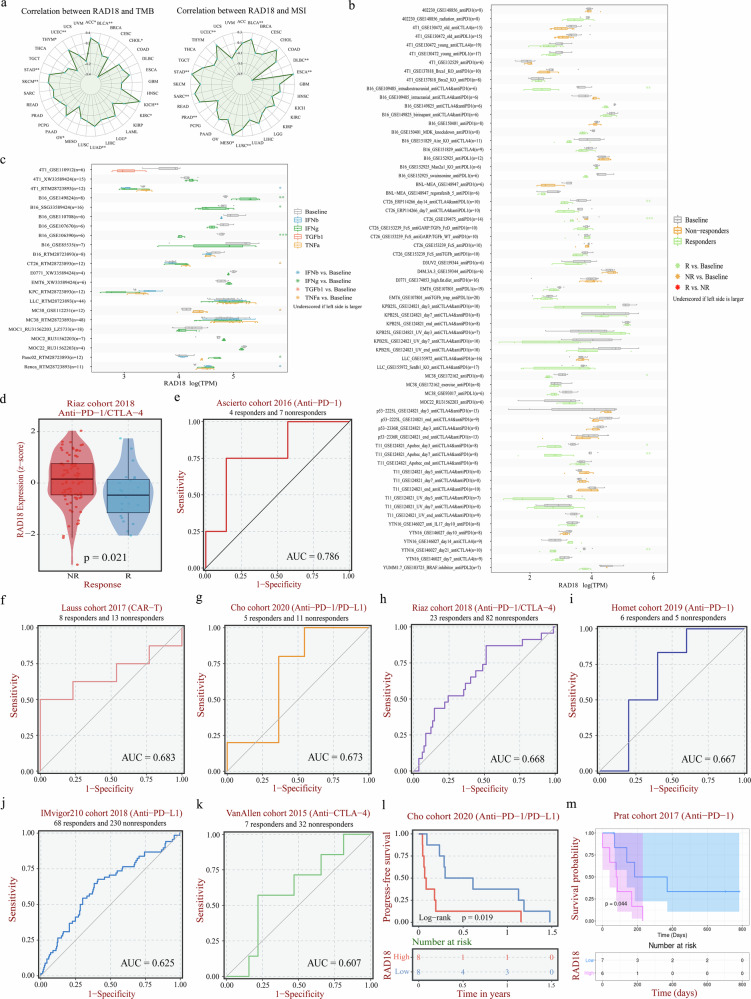
Correlation of RAD18 expression with immunotherapy response in a pan-cancer context. **a** The correlation between RAD18 expression and tumor mutational burden and microsatellite instability was evaluated by Spearman’s correlation analysis. **b** Based on the TISMO database, the expression differences of RAD18 in the in vivo cell lines of syngeneic mouse cancer models before and after immune checkpoint blockade (ICB) treatment and in different response groups were explored. **c** The expression differences of RAD18 in the in vitro cell lines of the syngeneic mouse cancer model before and after cytokine treatment. **d** Differences in RAD18 expression in patients who actively respond and those who do not respond to immunotherapy in the Riaz 2018 anti-PD1/CTLA4 cohort. **e**–**k** The predictive performance of RAD18 for ICB treatment response was evaluated based on the AUC values. **l**, **m** Difference in overall survival between RAD18 high-expresser and low-expresser subgroups in Cho cohort 2020 (anti-PD1/PDL1) (**l**) and Prat cohort 2017 (anti-PD1) (**m**). *p < 0.05; **p < 0.01; and ***p < 0.001.

To clarify the predictive role of RAD18 in the response to immunotherapy of different cancers, we first selected the predictive biomarkers TMB and MSI related to immunotherapy^25^ and evaluated their relationship with RAD18 expression. Figure. 8a reveals that RAD18 expression exhibits a positive correlation with TMB in ACC, BLCA, KICH, KIRC, LGG, LUAD, SKCM, STAD, and UCEC, whereas a negative correlation is observed in CHOL, OV, and THYM. Moreover, RAD18 expression was positively associated with MSI in BLCA, ESCA, LUSC, MESO, SARC, STAD, and UCEC, yet negatively associated with MSI in DLBC and PRAD (Fig. 8a).

Subsequently, we accessed the TISMO database and predicted the response of cancer individuals to immunotherapy drugs according to the expression level of RAD18. As depicted in Fig. 8b, after ICB treatment (anti-PD1, anti-PDL1, anti-CTLA4, anti-CTLA4 + anti-PD1/L1), RAD18 showed a significant downregulation trend in the response group of seven in vivo cell lines derived from syngeneic mouse tumor models (B16_GSE109485, CT26_ERP114266, CT26_GSE139475, MC38_GSE172162, T11_GSE124821_Apobec_day3, T11_GSE124821_Apobec_day7, YTN16_GSE146027). After cytokine treatment, RAD18 expression in the in vitro cell lines of eight syngeneic mouse tumor models also changed significantly (Fig. 8c). Among them, RAD18 expression was significantly downregulated in the 4T1_RTM28723893 cell line upon IFN-β treatment, and the B16_SSG33589424/GSE106390 cell line upon IFN-γ treatment. In contrast, RAD18 expression was significantly upregulated in B16_GSE149824 cell line upon IFN-γ treatment, CT26_RTM28723893 and MC38_GSE112251 cell lines upon TNF-α treatment, Panc02_RTM28723893 cell line upon TNF-γ treatment, and Renca_RTM28723893 cell line upon TNF-β treatment.

In human tumor immunotherapy cohorts, decreased RAD18 expression may be associated with improved immunotherapy effects. For example, in the Riaz 2018 anti-PD1/CTLA4 cohort, the expression level of the RAD18 gene was significantly lower in patients who showed a positive response to treatment compared to those who did not respond to immunotherapy (Fig. 8d). According to AUC values, the predictive accuracy of RAD18 expression for tumor immune response, ranked from high to low, was as follows: the Ascierto cohort in 2016 (with an AUC of 0.786), the Lauss cohort in 2017 (0.683), the Cho cohort in 2020 (0.673), the Riaz cohort in 2018 (0.668), the Homet cohort in 2019 (0.667), the IMvigor210 cohort in 2018 (0.625), and the VanAllen cohort in 2015 (0.607) (Fig. 8e–k). The median expression value of RAD18 divided patients in the tumor immunotherapy cohort into high-expresser and low-expresser subgroups. We found that in the 2020 Cho cohort (anti-PD1/PDL1) and the 2017 Prat cohort (anti-PD1), the OS of the RAD18 high-expresser subgroup was markedly inferior to that of the RAD18 low-expresser subgroup (Fig. 8l, m).

Ultimately, we assessed the association of RAD18 expression levels with the sensitivity to small-molecule drugs across the pan-cancer cohort (Supplementary Fig. 9). In the GDSC dataset, the drug sensitivity of Afatinib, Cetuximab, Gefitinib, and UNC1215 was strongly positively correlated with RAD18 mRNA expression, while the remaining 26 small molecule drugs were strongly negatively correlated with ADGRG6 expression (false detection rate (FDR) < 0.05). In the CTRP dataset, the top 30 drugs, including clofarabine and triazolothiadiazine, were strongly negatively correlated with RAD18 expression (FDR < 0.05). In summary, the expression status of RAD18 has an important and promising value in accurately predicting tumor response to immunotherapy and anti-tumor small molecule drugs.

### Identification of the carcinogenic role of RAD18 in cancer

**Fig. 9:**
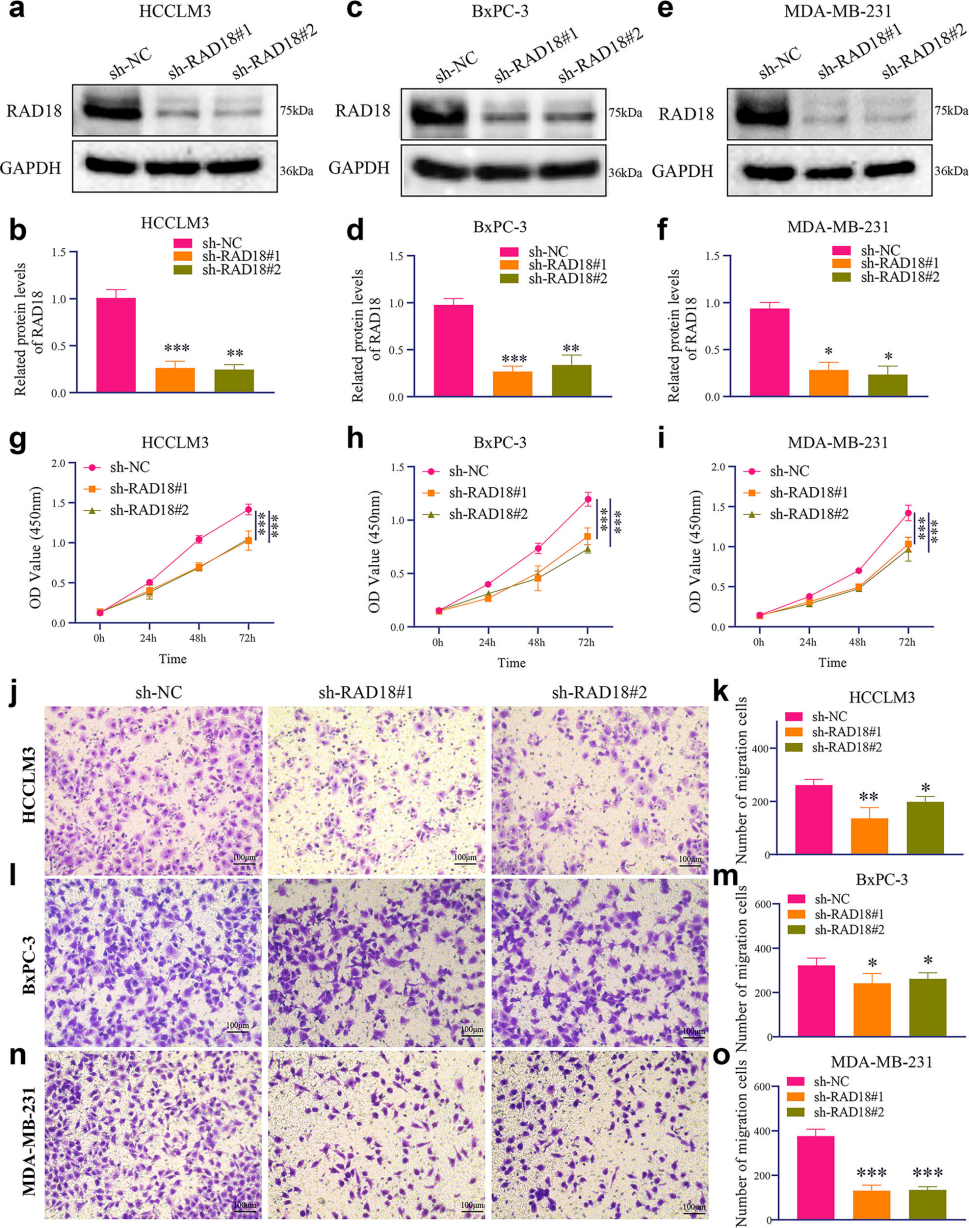
Silencing RAD18 expression inhibits LIHC, PAAD and Basal-like BRCA progression. **a**–**f** RAD18 expression was silenced in HCCLM3, BxPC-3, and MDA-MB-231 cells, and the silencing efficiency was verified by Western blot. **g**–**i** The difference in cell proliferation activity between the control and RAD18-inhibited groups in HCCLM3, BxPC-3, and MDA-MB-231 cell lines was detected by CCK-8 cell viability assay. **j**–**o** The difference in cell migration ability between the control and RAD18-inhibited groups in HCCLM3, BxPC-3, and MDA-MB-231 cell lines was analyzed by Transwell cell migration assay. *p < 0.05; **p < 0.01; ***p < 0.001.

**Fig. 10:**
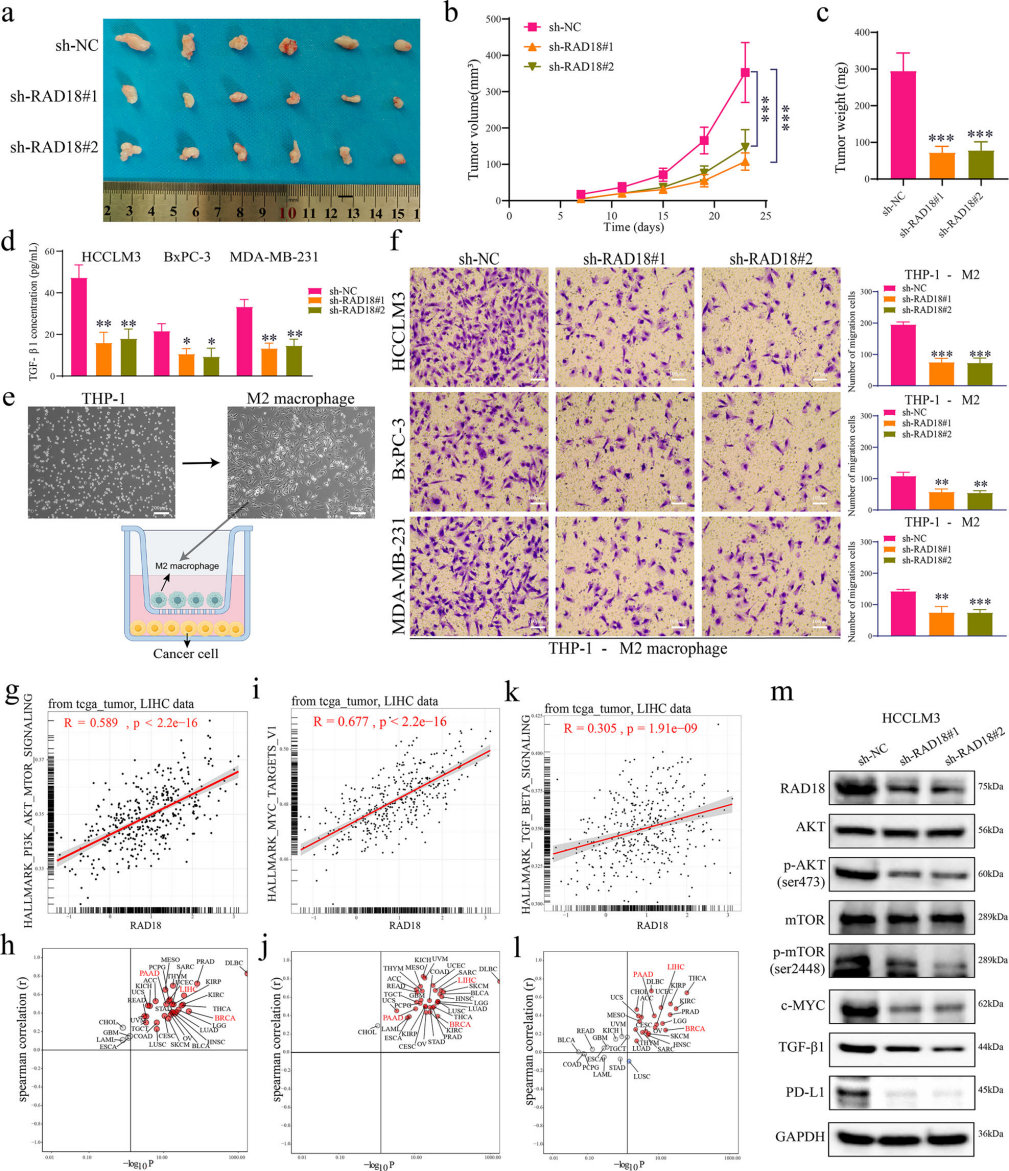
RAD18 drives tumor progression and immune evasion by activating the AKT/mTOR/c-MYC/TGF-β1/PD-L1 pathway. **a**–**c** In vivo xenograft tumor growth assay using HCCLM3 cells with stable RAD18 knockdown. **a** Representative images of excised tumors; (**b**) tumor volume growth curve; (**c**) tumor weight quantification (***P < 0.001). **d** ELISA was used to analyze the TGF-β1 content in the cell supernatant of HCCLM3, BxPC-3, and MDA-MB-231 cells 48 hours after RAD18 knockdown. (*p < 0.05, **p < 0.01). **e** Schematic illustration of THP-1 differentiation into M2 macrophages and the transwell co-culture system for assessing macrophage chemotaxis. By Figdraw. **f** Representative images and quantification of Transwell assays showing M2 macrophage migration toward HCCLM3, BxPC-3, and MDA-MB-231 cells with RAD18 knockdown (scale bar = 100 μm; *p < 0.05, ***p < 0.001). **g**–**l** Gene set enrichment analysis (GSEA) and correlation network analysis showing that RAD18 expression positively correlates with the PI3K/AKT/mTOR signaling pathway (**g**, **h**), MYC targets V1 (**i**, **j**), and TGF-β signaling pathway (**k**, **l**) across multiple tumor types (LIHC data shown as representative). **m** Western blot analysis demonstrating that RAD18 knockdown in HCCLM3 cells suppresses phosphorylation of AKT (Ser473) and mTOR (Ser2448), and downregulates the expression of c-MYC, TGF-β1, and PD-L1. GAPDH was used as the loading control.

Previous integrative analyses have demonstrated that RAD18 is highly expressed in LIHC, PAAD, and Basal-like breast cancer (BRCA), and its elevated expression is closely associated with poor prognosis; however, the underlying functional mechanisms remain largely unexplored. To further validate the oncogenic role of RAD18 in tumors, we performed a series of in vitro and in vivo experiments. As shown in Fig. 9a–f, we successfully knocked down RAD18 expression in HCCLM3, BxPC-3, and MDA-MB-231 cells using two independent shRNA constructs. Subsequent CCK-8 assays revealed that RAD18 knockdown significantly suppressed the proliferative activity of these cell lines compared with the sh-NC control group (Fig. 9g–i). Transwell migration assays further confirmed that RAD18 silencing markedly impaired the migratory capacity of HCCLM3, BxPC-3, and MDA-MB-231 cells (Fig. 9j–o). In vivo xenograft experiments using the HCCLM3 cell line demonstrated that tumors in the stable RAD18-knockdown groups (sh-RAD18#1 and sh-RAD18#2) exhibited significantly reduced volume and weight compared with the sh-NC control group (Fig. 10a–c).

Additionally, ELISA assays showed that silencing RAD18 expression markedly inhibited the secretion of TGF-β1 in HCCLM3, BxPC-3, and MDA-MB-231 cells relative to controls (Fig. 10d). Next, we induced THP-1 cells to differentiate into M2-type macrophages using a well-established protocol (Fig. 10e)^26,27^. Silencing RAD18 in HCCLM3, BxPC-3, and MDA-MB-231 cells significantly reduced the chemotactic migration of M2-type macrophages toward these tumor cells (Fig. 10f).

As illustrated in Fig. 10g–l, gene set enrichment analysis (GSEA) revealed that RAD18-correlated genes were significantly enriched in the PI3K/AKT/mTOR signaling pathway, MYC targets V1, and TGF-β pathway across most tumor types, including LIHC, PAAD, and BRCA. Notably, aberrant activation of these signaling cascades is closely implicated in tumor progression and immune regulation. Further Western blot analysis confirmed that silencing RAD18 in HCCLM3, BxPC-3, and MDA-MB-231 cells suppressed the phosphorylation levels of AKT and mTOR, and downregulated the expression of c-MYC, TGF-β1, and PD-L1 (Fig. 10m and Supplementary Fig. 10a, b).

Collectively, these findings establish that RAD18 acts as an oncogene in liver, pancreatic, and Basal-like breast cancers by activating the AKT/mTOR/c-MYC signaling axis to promote tumor cell proliferation, migration, and TGF-β1/PD-L1-mediated immune suppression, highlighting its potential as a prognostic biomarker and therapeutic target for these malignancies.

### RAD18 promotes tumor progression through the AKT/c-MYC pathway

**Fig. 11:**
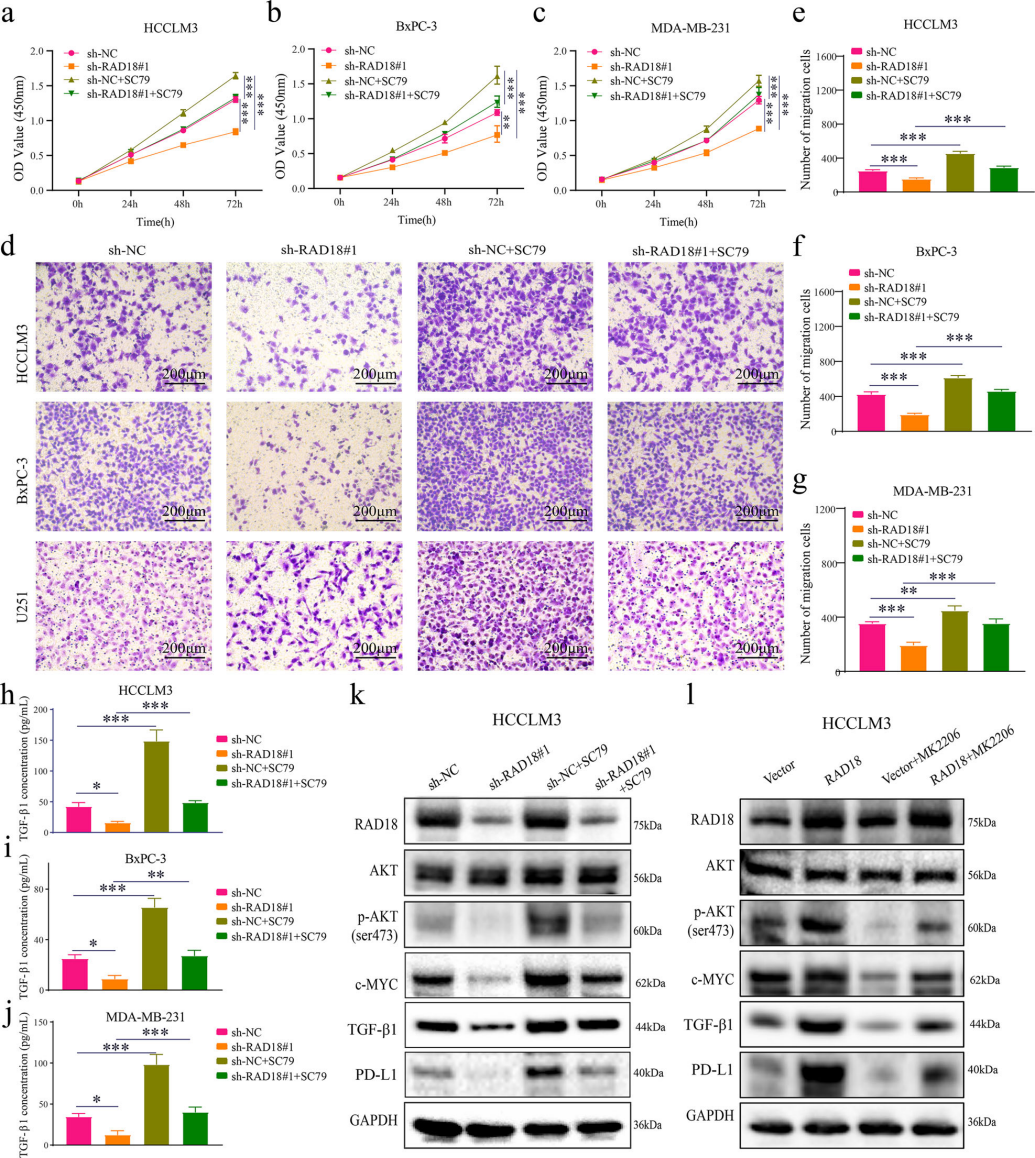
RAD18 promotes tumor cell malignant progression via the AKT/c-MYC Pathway. **a**–**c** CCK-8 assays showing that the AKT activator SC79 partially restores the proliferative capacity of HCCLM3 (**a**), BxPC-3 (**b**), and MDA-MB-231 (**c**) cells with RAD18 knockdown. **d** Representative images of Transwell migration assays for HCCLM3, BxPC-3, and MDA-MB-231 cells under the indicated treatments (scale bar = 200 μm). **e**–**g** Quantification of migrated cell numbers, demonstrating that SC79 reverses the migration inhibition caused by RAD18 knockdown. **h**–**j** ELISA assays confirming that SC79 rescues the reduction in TGF-β1 secretion induced by RAD18 knockdown in HCCLM3 (**h**), BxPC-3 (**i**), and MDA-MB-231 (**j**) cells. **k** Western blot analysis showing that SC79 partially restores the expression of p-AKT (Ser473), c-MYC, TGF-β1, and PD-L1 in RAD18-knockdown HCCLM3 cells. **l** Western blot analysis demonstrating that the AKT inhibitor MK2206 partially abrogates the upregulation of p-AKT (Ser473), c-MYC, TGF-β1, and PD-L1 induced by RAD18 overexpression in HCCLM3 cells. GAPDH was used as the loading control. *p < 0.05, **p < 0.01, ***p < 0.001.

To verify whether RAD18 regulates tumor cell proliferation and migration through the AKT/c-MYC pathway, we performed rescue experiments using the AKT activator SC79. CCK-8 assays (Fig. 11a–c) showed that RAD18 knockdown (sh-RAD18#1) significantly inhibited cell proliferation within 72 h, whereas AKT activation via SC79 partially restored the proliferative capacity of HCCLM3, BxPC-3, and MDA-MB-231 cells. Transwell migration assays (Fig. 11d–g) demonstrated that RAD18 knockdown markedly suppressed cell migration, and SC79 treatment reversed this migratory inhibition in the three cell lines. ELISA assays revealed that SC79 could rescue the reduction in TGF-β1 secretion induced by RAD18 knockdown in HCCLM3, BxPC-3, and MDA-MB-231 cells (Fig. 11h–j). Western blot analysis (Fig. 11k and Supplementary Fig. 10c,d) showed that RAD18 knockdown obviously decreased the expression levels of p-AKT (Ser473), c-MYC, TGF-β1, and PD-L1, which were partially restored by SC79. Conversely, RAD18-overexpressing cells were treated with the AKT inhibitor MK2206. We found that RAD18 overexpression significantly upregulated the expression levels of p-AKT (Ser473), c-MYC, TGF-β1, and PD-L1 in HCCLM3, BxPC-3, and MDA-MB-231 cells, and these upregulations were partially abrogated by MK2206 (Fig. 11l and Supplementary Fig. 10e, f). Consistent with the above findings, ELISA assays further confirmed that MK2206 treatment significantly reversed the elevated TGF-β1 secretion triggered by RAD18 overexpression in the three cell lines (Supplementary Fig. 10g–i).

Collectively, these rescue experiments demonstrate that RAD18 drives the malignant progression of LIHC, PAAD, and Basal-like BRCA partially through the AKT/c-MYC pathway, solidifying the pivotal role of this signaling axis in RAD18-mediated oncogenesis.

### Clinical relevance of RAD18 in triple-negative breast cancer and its role in anti-PD-1 immunotherapy resistance

Collectively, our in vitro and in vivo mechanistic studies established that RAD18 drives malignant progression and immune suppression in LIHC, PAAD, and Basal-like BRCA via the AKT/c-MYC/TGF-β1/PD-L1 signaling axis. To translate these findings into clinical relevance, we evaluated RAD18 expression in clinical triple-negative breast cancer (TNBC) specimens and assessed its correlation with patient prognosis and immunotherapy response.

**Fig. 12:**
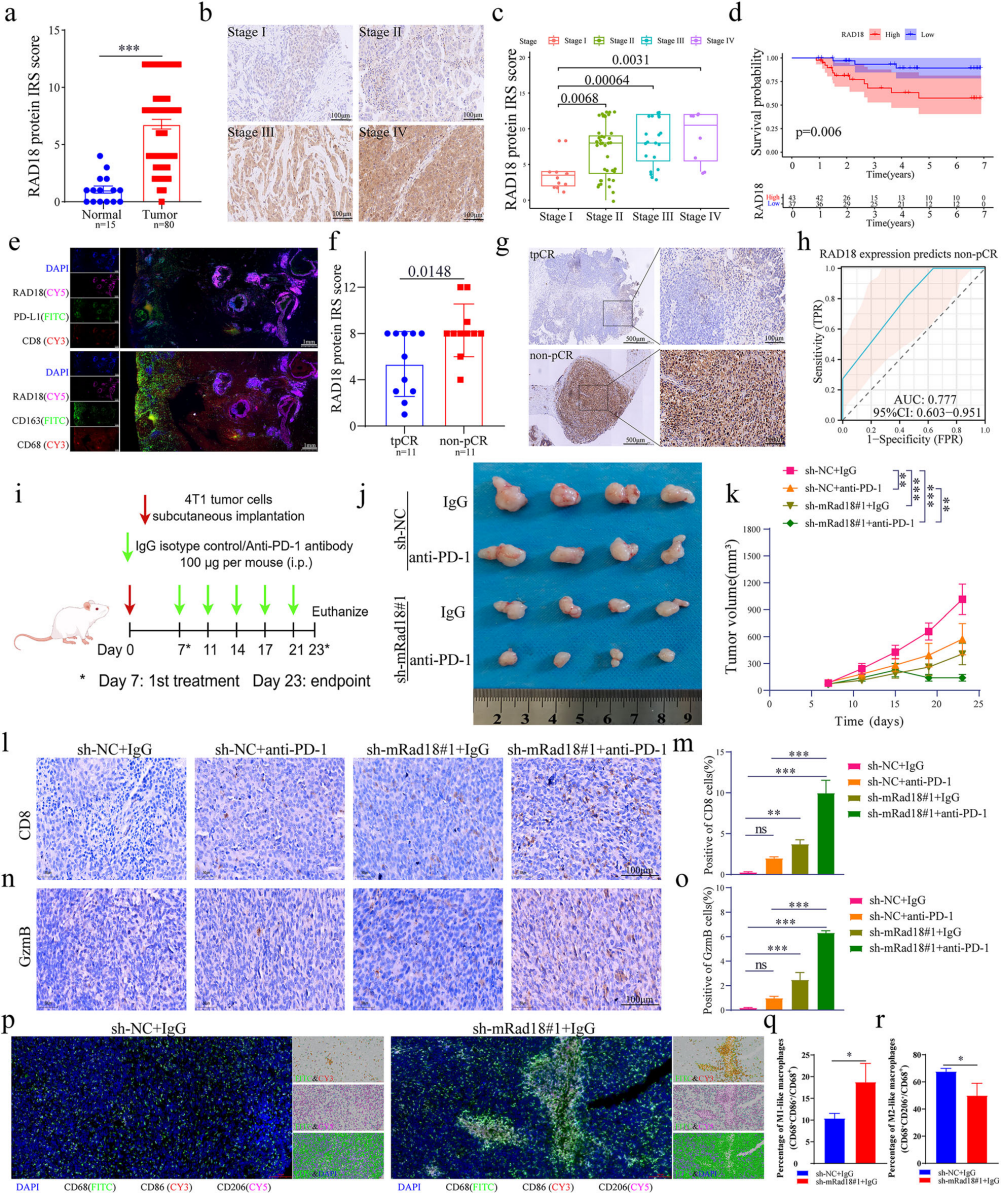
High RAD18 expression is associated with tumor progression and resistance to anti-PD-1 immunotherapy in triple-negative BRCA(TNBC). **a** Box plot showing significantly higher RAD18 protein IRS scores in TNBC tumor tissues compared with normal adjacent tissues. **b** Representative IHC images of RAD18 staining in TNBC tissues from Stage I to Stage IV (scale bar = 100 μm). **c** Box plot demonstrating that RAD18 protein expression increases with advanced tumor stage. **d** Kaplan–Meier survival curve showing that high RAD18 expression correlates with shorter overall survival in TNBC patients. **e** Multiplex immunofluorescence staining showing co-localization of RAD18, PD-L1, CD8, CD163, and CD68 in TNBC tissues (scale bar = 1 mm). **f** Box plot showing higher RAD18 protein IRS scores in non-pCR breast cancer patients compared with pCR patients. tpCR: Total pathological Complete Response; non-pCR: non-pathological Complete Response. **g** Representative IHC images of RAD18 staining in pCR and non-pCR TNBC tissues. **h** ROC curve demonstrating that RAD18 expression predicts non-pCR in TNBC (AUC = 0.777; 95%CI:0.603–0.951). **i** Schematic illustration of the 4T1 syngeneic mouse model and treatment schedule with anti-PD-1 antibody. By Figdraw. **j**, **k** In vivo 4T1 tumor growth assay showing that RAD18 knockdown (sh-mRad18#1) enhances the anti-tumor efficacy of anti-PD-1 immunotherapy. **j** Representative images of excised tumors; (**k**) tumor volume growth curve. **l**–**o** IHC staining and quantification showing that combined RAD18 knockdown and anti-PD-1 treatment increases the infiltration of CD8⁺ T cells (**l**, **m**) and GzmB⁺ cytotoxic T cells (**n**, **o**) in 4T1 tumors. **p**–**r** Multiplex immunofluorescence staining and quantification showing that RAD18 knockdown reduces the percentage of M2-like macrophages (CD68⁺CD206⁺) and increases the percentage of M1-like macrophages (CD68⁺CD86⁺) in 4T1 tumors. *p < 0.05, **p < 0.01, ***p < 0.001.

Immunohistochemical (IHC) analysis revealed that RAD18 protein expression (quantified by immunoreactive score, IRS) was significantly elevated in TNBC tumor tissues compared with matched normal adjacent tissues (Fig. 12a). RAD18 staining intensity gradually increased with advanced tumor stage (Fig. 12b, c), and high RAD18 expression was significantly associated with shorter overall survival in TNBC patients (Fig. 12d). Multiplex immunofluorescence staining (Fig. 12e) demonstrated that RAD18 was extensively co-localized with PD-L1 in TNBC tissues, and their expression levels displayed a positive correlation. Strikingly, regions with high RAD18 expression exhibited markedly reduced or absent CD8⁺ T cell infiltration, indicating a negative correlation between RAD18 expression and intratumoral CD8⁺ T cell abundance. Furthermore, the distribution of CD68⁺CD163⁺ M2-like tumor-associated macrophages was highly consistent with RAD18-high areas in TNBC specimens (Fig. 12e).

Given the critical role of RAD18 in remodeling the tumor immune microenvironment, we further investigated its clinical predictive value in a well-defined TNBC cohort receiving neoadjuvant immunotherapy containing anti-PD-1 inhibitors. Notably, RAD18 IRS scores were significantly higher in tumor specimens from patients with non-pathological complete response (non-pCR) than those from patients achieving total pathological complete response (tpCR; Fig. 12f, g). ROC curve analysis further validated that RAD18 expression could effectively predict non-pCR status in TNBC (AUC = 0.777; 95% CI: 0.603–0.951; Fig. 12h). To further verify the role of RAD18 in regulating immunotherapy efficacy in vivo, we screened and established stable RAD18-knockdown 4T1 cell lines, and Western blot analysis confirmed the knockdown efficiency of sh-mRad18 constructs (Supplementary Fig. 10j). The sh-mRad18#1 cell line with optimal silencing efficiency was then selected for subsequent syngeneic mouse experiments. In a 4T1 syngeneic TNBC mouse model (Fig. 12i), RAD18 knockdown (sh-mRad18#1) alone or in combination with anti-PD-1 antibody significantly suppressed tumor growth, with the combination treatment exerting the strongest anti-tumor effect (Fig. 12j, k). IHC staining further revealed that the combined treatment markedly increased the infiltration of CD8⁺ cytotoxic T cells and Granzyme B (GzmB)⁺ functional T cells (Fig. 12l–o). Additionally, immunofluorescence analysis of control and RAD18-knockdown tumors showed that RAD18 silencing significantly shifted macrophage polarization toward the anti-tumor M1-like phenotype (CD68⁺CD86⁺) and decreased the proportion of pro-tumor M2-like tumor-associated macrophages (CD68⁺CD206⁺; Fig. 12p–r). Collectively, these clinical and in vivo findings highlight that high RAD18 expression correlates with aggressive tumor phenotypes and inferior immunotherapy response in TNBC, and targeting RAD18 represents a promising strategy to enhance the efficacy of anti-PD-1 immunotherapy by remodeling the tumor immune microenvironment.

## Discussion

Although remarkable headway has been made in combating cancer, the advancement of conventional chemotherapy agents remains constrained by the research model. This model predominantly relies on the tumor’s location and histological subtype instead of specific genetic mutations. Although new gene and immune-targeted drugs use biomarkers as targets, they are difficult to clinically transform in rare or ultra-rare tumors, and decades of data accumulation are required^16^. Basket trials provide innovative paths, such as targeting BRAF V600E mutations, including patients with this mutation in different cancer types, and treating them with dabrafenib combined with trametinib. If there is a response, the summary of multi-cancer data can promote the application of therapies across cancer types^28^. In this context, tissue-agnostic therapy has become a breakthrough direction for precision oncology. It targets the core genetic drivers of tumors, provides new options for patients with rare cancers and drug resistance, and accelerates drug development^16^. However, the U.S. Food and Drug Administration has only approved six tissue-agnostic target drugs for advanced solid tumors, and there is an urgent need to discover and verify more potential pan-cancer carcinogenic drivers to expand their application.

RAD18-mediated monoubiquitination of PCNA promotes the employment of error-prone polymerases (e.g., Y-family polymerases), significantly accelerating the risk of mutagenesis and carcinogenesis^29^. Earlier studies have documented the expression profile and function of RAD18 within melanoma^30^, ESCC^7^, GC^8^, and glioma^11^. They found that RAD18 expression was elevated in these cancers and was associated with tumor progression and worse prognosis. However, these conclusions were limited to limited and specific cancer types. This investigation is the pioneer in thoroughly assessing the expression levels and clinical significance of RAD18 across a pan-cancer dataset. We observed that RAD18 exhibited elevated expression in the majority of cancer tissues (22 types) and was frequently linked to an unfavorable prognosis. In particular, the RAD18 high-expressor subgroup independently predicted poor prognosis in ACC (HR: 4.011), MESO (HR: 2.568), LGG (HR: 1.885), LIHC (HR: 1.726), and PAAD (HR: 1.599). In addition, RAD18 showed excellent diagnostic value in more than ten malignant tumors such as CHOL, GBM, and SARC, among others (all AUC values > 0.9). Pan-cancer biological function results indicated that RAD18 predominantly participated in cell-cycle and proliferation-related pathways. This discovery offers crucial insights for comprehending and validating the latent mechanisms of RAD18 in carcinogenesis and cancer progression. Furthermore, in several immunotherapy cohorts, patients with high RAD18 expression had poorer immunotherapy outcomes than those with low RAD18 expression, suggesting that high RAD18 expression may promote an immunosuppressive microenvironment. In vitro and in vivo experiments further confirmed that silencing RAD18 can inhibit the malignant progression of LIHC cells through the PI3K/AKT/mTOR signaling pathway. In addition, indirect co-culture experiments showed that inhibiting RAD18 expression reduced chemotaxis of M2 macrophages. Based on these results, this study aims to provide theoretical support for RAD18 as a prognostic and immunotherapy efficacy indicator for various malignant tumors in the field of tumor immunology.

Previously, researchers ascribed the aberrant expression of malignancy-associated genes to the following genetic alterations: (1) mutations in gene regulatory regions, (2) abnormal expression of transcription factors involved in gene regulation, (3) gene amplification, and (4) insertion of viral promoters upstream of genes^31^. In this study, we noticed that about 60% of cancers overexpressing RAD18 had genetic alterations. Among these genetic changes in these cancers, amplification and mutation of RAD18 were the predominant forms, the former mainly seen in BLCA, OV, BRCA, LGG, GBM, LIHC, and the latter mainly in SKCM, COAD, LUSC, HNSC, LUAD, and KIRP. DNA methylation represents the epigenetic modification that has undergone the most extensive investigation. It refers to the transfer of the methyl group on the cofactor S-adenosyl-L-methionine to the fifth carbon atom of the cytosine residue in DNA with the help of DNA methyltransferase, thereby generating 5-methylcytosine associated with transcriptional repression of transposable elements^32^. In cancer cells, DNA methylation is characterized by genome-wide hypomethylation and site-specific hypermethylation (specifically, promoter-region CpG island hypermethylation of tumor suppressor genes)^21^. These methylation patterns are closely associated with the carcinogenic process. In this study, about 45% of cancers that overexpressed RAD18 showed abnormal DNA methylation. Among them, RAD18 showed significant DNA hypomethylation in cancer tissues of BLCA, PAAD, CESC, COAD, LUAD, PAAD, and READ. These findings suggest that the upregulation of RAD18 in multiple cancers may be related to its genetic variation patterns and epigenetic modifications in pan-cancer.

By analyzing and comparing the results of Hallmark gene set enrichment analysis, we identified that cell cycle- and proliferation-related pathways are among the main pathways enriched by RAD18 in various cancer types, specifically including Myc Targets V1, E2F targets, G2M checkpoint, and mitotic spindle. A previous study reported that CENPF, which is upregulated in lung adenocarcinoma, correlates with unfavorable clinical prognoses and an immunosuppressive state among individuals with lung adenocarcinoma, and is significantly enriched in the progesterone-mediated oocyte maturation pathway^33^. In addition, E2F targets have been demonstrated to facilitate prostate cancer metastasis, along with tumor growth and treatment resistance in estrogen receptor-positive breast cancer^34,35^. G2/M cell cycle arrest induced by BUB1 depletion attenuates the invasive function of malignant pleural mesothelioma cells^36^. Excessive activation of the spindle assembly checkpoint mediated by YY2/BUB3 inhibits colorectal cancer growth by triggering mitotic delay^37^. In the present study, we systematically uncovered the critical role of RAD18 in promoting malignant progression and immune suppression across LIHC, PAAD, and TNBC, and further validated that RAD18 executes these oncogenic functions partially through the AKT/mTOR/c-MYC signaling pathway via a series of rescue experiments. Our findings not only expand the current understanding of RAD18-mediated tumor pathogenesis beyond its canonical role in DNA damage repair, but also establish RAD18 as a novel predictive biomarker for immunotherapy response and a promising therapeutic target to overcome anti-PD-1 resistance in hard-to-treat solid tumors.

As a breakthrough in traditional anti-cancer treatment, immunotherapy has brought longer survival benefits to a limited number of cancer patients. Nevertheless, the capacity of neoplastic cells to evade immune system monitoring and assault has substantially constrained the clinical effectiveness of immunotherapy^38^. Previous reports indicate that deficiencies in DNA damage repair can boost the effectiveness of ICB by augmenting genomic instability, increasing the level of new antigens and tumor immunogenicity, and promoting immune response activation and immune environment remodeling^38–40^. Given the TLS function of RAD18, which increases the probability of mutagenesis due to low fidelity of DNA repair, it may have potential value in predicting immunotherapy response. Within the scope of this investigation, it was ascertained that there was a notable inverse correlation between RAD18 and the infiltration levels of diverse immune cell populations, such as B cells, CD8 T cells, Cytotoxic cells, NK CD56bright cells, NK CD56dim cells, NK cells, and Th1 cells, which are considered to have considerable anti-tumor immune effects^41–47^. Conversely, RAD18 shows a marked positive association with the infiltration extent of immune cell types, including Th2. Compared with Th1 cells, which have a clear anti-cancer immune effect, Th2 cells have more diverse functions. Given the antagonistic regulatory relationship between the differentiation pathways of Th1 and Th2 cells, Th2 cells exhibit pro-tumor effects in cancer models where Th1-mediated anti-tumor immunity is prevalent^48^. In addition, in this study, we found that silencing RAD18 inhiblimited the recruitment of M2 macrophages - this result further confirmed that RAD18 plays a pro-carcinogenic role in the tumor immune microenvironment by regulating immune cell infiltration.

Previously, only a handful of studies have either directly or indirectly probed into the association of RAD18 with anti-tumor immune responses. For example, the transcription factor E2F7 was reported to inhibit the killing of colorectal cancer cells by NK cells by promoting RAD18 transcription^49^. Knockdown of RAD18 expression significantly reduced Dox-mediated DDT, promoted immunogenic cell death, and further enhanced the anti-tumor effect against osteosarcoma^50^. Interestingly, a study by Ma et al. found that LINC01116 stabilizes EWSR1 by preventing RAD18-mediated ubiquitination. EWSR1 causes cancer cells to have more linoleic acid (a growth-promoting nutrient) than T cells, resulting in the impairment of T-cell function and promoting LIHC malignant progression^51^. In this context, RAD18 appears to exert an indirect influence on anti-tumor immune responses. We observed that the improvement of immunotherapy response was significantly associated with the downregulation of RAD18 expression, both in mouse cell lines and human immunotherapy cohorts. Specifically, RAD18 expression showed a significant downregulation trend in the response group of seven in vivo cell lines derived from syngeneic mouse tumor models. The anti-PD1/CTLA4 treatment cohort study conducted by Riaz in 2018 also showed that patients who responded positively to immunotherapy had significantly lower RAD18 gene expression levels in vivo than those who did not respond^52^. In addition, in several immunotherapy cohorts, the OS of the RAD18 high-expresser subgroup was significantly worse than that of the RAD18 low-expresser subgroup. Multiplex immunofluorescence staining of clinical TNBC specimens revealed that RAD18 was positively co-localized with PD-L1 expression, and inversely correlated with intratumoral CD8⁺ cytotoxic T cell infiltration. Additionally, RAD18-high regions exhibited enriched M2-like tumor-associated macrophages, which are known to suppress anti-tumor immunity and foster immune evasion. Consistent with these clinical observations, in vivo experiments using a syngeneic 4T1 mouse model showed that RAD18 knockdown alone not only inhibited tumor growth, but also shifted macrophage polarization from pro-tumor M2 to anti-tumor M1 phenotype; notably, combining RAD18 silencing with anti-PD-1 antibody further synergistically enhanced CD8⁺ T cell infiltration and cytotoxicity, resulting in superior anti-tumor efficacy compared with monotherapy. The clinical translational value of our findings is further underscored by the analysis of a TNBC cohort receiving neoadjuvant anti-PD-1-based immunotherapy. We observed that RAD18 expression was significantly upregulated in TNBC tissues compared with adjacent normal tissues, and its expression level positively correlated with advanced tumor stage and worse overall survival. Moreover, high RAD18 expression was strongly associated with non-pCR status after neoadjuvant immunotherapy, and ROC curve analysis confirmed its predictive value for treatment response. These clinical data corroborate our preclinical findings, suggesting that RAD18 can serve as a reliable biomarker to stratify TNBC patients who may benefit from anti-PD-1 immunotherapy, and targeting RAD18 represents a viable strategy to sensitize resistant tumors to immune checkpoint blockade.

Despite the above findings, several limitations in this study should be acknowledged. Notably, our results confirm that the AKT/mTOR/c-MYC pathway only partially mediates RAD18-driven oncogenicity and immune suppression, and a clear molecular bridge linking the nuclear DNA repair protein RAD18 to cytoplasmic PI3K/AKT/mTOR kinase signaling remains to be elucidated. This critical mechanistic gap hinders the full comprehension of how RAD18 shuttles between the nucleus and cytoplasm to transduce oncogenic signals, and further studies are urgently needed to identify the intermediate adaptor proteins, post-translational modifications, or non-canonical interactors that connect RAD18 to the cytoplasmic kinase cascade. Moreover, future investigations are warranted to explore the upstream regulatory factors of RAD18 across different cancer types and potential crosstalk between RAD18 and other oncogenic or immune-related pathways, to establish a comprehensive regulatory network of RAD18 in tumor progression and immune evasion.

## Methods

### Assessment of RAD18 expression level, localization, and clinical features

RNA-seq datasets from the TCGA and GTEx databases were retrieved via the UCSC Xena platform^53^. After the Log2 (TPM + 1) transformation, we compared the mRNA expression disparity of RAD18 between normal and cancerous tissues. The subcellular localization profile and immunofluorescence images of RAD18 were obtained from the Genecards database and the Human Protein Atlas (HPA)^54^. The association between the mRNA expression of RAD18 and clinical grades and stages of the pan-cancer cohort was evaluated using the TISIDB web server^55^.

### Analysis of the genetic variation landscape and methylation level of RAD18

The genetic alteration types and gene mutation landscape of RAD18 across diverse cancers were visualized by cBioPortal^56^ and the Sangerbox platform, respectively. The difference in DNA methylation pattern at the RAD18 promoter between malignant and non-malignant tissues was analyzed and displayed using the UALCAN online tool^57,58^.

### Prognostic correlation analysis and diagnostic efficacy evaluation of RAD18 expression in pan-cancer

Diverse prognostic data related to RAD18 across the pan-cancer spectrum, such as overall survival (OS), disease-specific survival (DSS), and progression-free interval (PFI), were sourced from the research conducted by Liu et al^22^. A Cox regression analysis was carried out on the prognostic dataset according to the median RAD18 expression level, and a prognostic heat map was generated with the use of the ggplot2 package. Cancer patients were stratified into RAD18 high-expresser and low-expresser subgroups according to the median RAD18 expression level. Subsequently, the prognostic disparity between the two cohorts was assessed via Kaplan-Meier survival curve analysis and the log-rank significance test. The prognostic role of RAD18 in breast cancer and its various subtypes was analyzed online using the Kaplan-Meier Plotter tool. The capacity of RAD18 for differentiating tumors from normal samples was gauged by determining the area under the receiver operating characteristic (ROC) curve (AUC)^59^. In this study, an AUC value greater than 0.75 represented good diagnostic performance^60,61^.

### Single-cell and spatial transcriptome data analysis

In this study, cross-cancer single-cell transcriptome datasets from the TISCH database were integrated, and a cross-cancer single-cell expression heatmap was generated using the pheatmap tool. Z-score normalization was used in data preprocessing steps. Sample similarity was evaluated via Euclidean distance matrices, and the Ward hierarchical clustering algorithm was utilized to refine the topological structure of rows and columns. This multi-dimensional visualization method effectively deciphered the specific expression profiles of cell subsets within the tumor microenvironment, thereby visually illustrating RAD18 expression.

CancerSEA is a dedicated database for in-depth analysis of various functional phenotypes of cancer cells at the single-cell level^62^. To assess the activity of specific pathways by integrating signature gene expression, 14 functional state gene sets were calculated using the zscore parameter in the R package GSVA, resulting in combined z-scores. These scores were further standardized via R’s scale function to define gene set scores, and Pearson’s correlation between RAD18 expression and each gene set score was determined.

Adopting the approach from prior studies^63^, 10xVisium sequencing data were merged to construct a pan-cancer spatial transcriptomic landscape. After profiling the cell type of each microregion, these regions were labeled according to their primary cell type based on cell type proportions. The one-to-one correspondence between the naming of spatial transcriptomics sections and the original studies is available at: https://grswsci.top/static/Sparkle_st.html. Subsequently, the average RAD18 expression in microregions of different cell types within each section was examined. To improve cross-section comparability, Z-score standardization was performed using R’s scale function. Finally, the pheatmap package was employed for visualizing standardized data, offering a clear display of RAD18’s expression pattern.

### Assessment of RAD18’s biological function in the context of pan-cancer

With the help of the GTBA platform, we performed Hallmarks enrichment analysis of RAD18 in 33 cancer types and drew heat maps to explore significantly enriched gene sets or signaling pathways. Count plots were also drawn to show the absolute enrichment rate of specific gene set items.

### Evaluation of the correlation between RAD18 expression and pan-cancer immune landscape, immunotherapy response, and drug sensitivity

The “ESTIMATE” package^64^ was used to evaluate the correlation between RAD18 expression and tumor immune score and stromal score. The relationship between RAD18 expression and the abundance of 24 immune cell infiltrations^65^ was further evaluated by the ssGSEA algorithm^66^. The relationship between RAD18 expression and immune checkpoint gene expression, tumor mutational burden (TMB), and microsatellite instability (MSI) was evaluated by employing Spearman’s correlation analysis. Moreover, the TISMO database was accessed to explore RAD18 expression disparities in syngeneic mouse tumor cells pre- and post-ICIB and cytokine treatment, as well as across different response groups^19^. Across multiple immunotherapy cohorts (GSE67501, GSE100797, GSE126044, GSE91061, GSE111636), we examined how RAD18 expression influences cancer patients’ survival time and its potential to forecast immunotherapy response. We also explored the relationship between the drug sensitivity of the top 30 genes in the CTRP or GDSC databases and RAD18 expression using the GSCA database.

### Patients and tissue samples

For patients with pathologically confirmed primary triple-negative breast cancer (TNBC), surgically resected tumor and adjacent non-tumor tissues were collected and paraffin-embedded, and their clinical and prognostic information was retrospectively collected and followed up. Written informed consent was obtained from all patients prior to sample collection and data acquisition. This cohort was utilized to analyze the association between RAD18 expression and TNBC stage, clinical outcomes, as well as immune marker profiles.

The immunotherapy cohort comprised patients with pathologically confirmed primary TNBC diagnosed by core needle biopsy. Puncture biopsy specimens were collected and paraffin-embedded, and the efficacy of neoadjuvant immunotherapy was evaluated during follow-up. This cohort was used to investigate the correlation between RAD18 expression and immunotherapeutic response.

This study did not include patients under the age of 16. The study protocol was approved by the Ethics Committee of the Second Xiangya Hospital of Central South University (Approval No.: 20230314), and all procedures were conducted in accordance with the Declaration of Helsinki.

### In vitro experiments

HCCLM3 (SCSP-5093, Cell Bank of the Chinese Academy of Sciences), BxPC-3 (CL-0042, Procell, China) MDA-MB-231(CL-0150B, Procell, China), THP-1 (CL-0233, Procell, China) and 4T1 (CL-0007, Procell, China) were grown in DMEM or RPMI-1640 medium fortified with 10% fetal bovine serum. SC79 (HY-18749, MedChemExpress) and MK-2206 dihydrochloride (SJ-MX0056, Sparkjade) were used for in vitro experiments. In cell function experiments, Lipofectamine 2000 (Invitrogen) was used to transfect short hairpin RNA (shRNA) into cells for downregulating RAD18 expression. For in vivo experiments, to obtain HCCLM3 and 4T1 cell lines with stable RAD18 knockdown, lentivirus-packaged shRNA was employed, with non-targeting negative control shRNA as the control. Transfected cells were selected with puromycin (Beyotime, Shanghai, China), and stable cell lines were chosen for subsequent in vivo experiments. The shRNAs targeting human and murine RAD18 genes, as well as the corresponding overexpression plasmids for human RAD18, were synthesized by Focus Bioscience Co., Ltd (Shanghai, China). Total protein extraction and Western blotting protocols have been described in the literature^67,68^. Antibodies used in this study included anti-RAD18 (Proteintech, 18333-1-AP; 1:1000), anti-AKT (Proteintech, 10176-2-AP; 1:2000), anti-p-AKT(Ser473) (Proteintech, 66444-1-Ig; 1:2000), anti-mTOR (Proteintech, 66888-1-Ig; 1:5000), anti-p-mTOR(Ser2448) (Proteintech, 67778-1-Ig; 1:2000), anti-PD-L1 (Proteintech, 66248-1-Ig; 1:5000), anti-c-MYC(Proteintech, 10828-1-AP; 1:10000), anti-TGF-β1 (Proteintech, 26155-1-AP; 1:500), and anti-GAPDH (Proteintech, 60004-1-Ig; 1:10,000).

As previously reported^69,70^, we employed the cell counting kit-8 (CCK-8) for detecting cell proliferation alterations, conducted a transwell migration assay to measure cell migration changes. The cell densities for the CCK-8 assay and transwell assay were 5 × 10³ and 3 × 10⁴ cells per well, respectively.

### ELISA assay

Human TGF-β1 concentrations were quantified using a commercial ELISA Kit (KE00002, Proteintech) following the manufacturer’s instructions. Briefly, samples and serially diluted standards were added to pre-coated wells in duplicate and incubated at 37 °C. After washing off unbound reagents, biotinylated detection antibody and HRP-conjugated streptavidin were added sequentially for incubation. The color reaction was developed with TMB substrate and terminated with stop solution. Absorbance was measured at 450 nm with a reference wavelength of 630 nm via a microplate reader, and TGF-β1 levels were calculated based on the standard curve.

### M2 macrophage migration assay

THP-1 cells were differentiated into macrophages by induction with 100 ng/mL phorbol 12-myristate 13-acetate (PMA; Sigma, USA). Subsequently, the cells were incubated with 20 ng/mL IL-4 (abs04698; absin, China) and 20 ng/mL IL-13 (abs04079; absin, China) for 48 h to induce M2 polarization. M2 macrophages were collected and resuspended in serum-free RPMI-1640 medium. A total of 1.0 × 10⁵ cells (300 μL) were seeded into the upper chamber of a Transwell insert (8 μm pore size; Corning, NY, USA). For the lower chamber, 700 μL of DMEM medium containing 10% FBS was added, along with 1.0 × 10⁵ HCCLM3 or HuH-7 cells. After 48 h of incubation, cells in the upper chamber were fixed with 4% paraformaldehyde (15 min) and stained with 0.1% crystal violet (15 min). Migrated M2 macrophages were counted in three randomly selected fields of each membrane.

### Animal experiments

This animal experimental protocol was approved by the Animal Ethics Committee of The Second Xiangya Hospital of Central South University (Approval No.: 20251345) and conducted in strict accordance with the Guidelines for the Care and Use of Laboratory Animals. Inclusion criteria were as follows: 6-week-old SPF-grade male nude mice, weighing 20–25 g, with normal mental status and free of infectious diseases; no animals were excluded during the experiment. After purchase, the mice were housed in an SPF animal facility for a one-week acclimatization period. The sample size was determined with reference to commonly used sample sizes in previous literature. Using a random number table method, the nude mice were randomly divided into 3 groups. Stable HCCLM3 knockdown cell lines were established using lentivirus-packaged shRNA plasmids, and positive cells were obtained via puromycin selection. Cells from the control group (sh-NC) and stable knockdown groups (sh-RAD18#1, sh-RAD18#2) in the logarithmic growth phase were collected separately and resuspended in PBS to a concentration of 1 × 10⁷ cells/mL. Each nude mouse was subcutaneously injected with 100 μL of the aforementioned cell suspension into the dorsal region. Tumor growth was measured starting from day 7 post-inoculation, with measurements taken once every 4 days. The formula for calculating tumor volume was: Tumor volume = long diameter × (short diameter)² × 0.52. At the experimental endpoint, the nude mice were euthanized by carbon dioxide (CO₂) inhalation followed by cervical dislocation. The tumors were dissected, rinsed with PBS to remove surface blood, and then weighed. The animal experiment of combined immunotherapy was performed as previously described^71^. Briefly, 4T1 cells stably transfected with sh-NC or sh-Rad18 were subcutaneously injected into female BALB/c mice. When tumor volumes reached 50–100 mm³, mice were randomly divided into four groups and treated with anti-PD-1 antibody or IgG control, respectively. At the experimental endpoint, mice were sacrificed by CO₂ inhalation followed by cervical dislocation, and tumors were excised, weighed, and subjected to subsequent analyses.

### Multiplex immunofluorescence staining and image analysis

Formalin-fixed paraffin-embedded (FFPE) tissue sections (4 μm) were deparaffinized, rehydrated, and subjected to antigen retrieval in EDTA buffer (pH 9.0) at 95 °C for 20 min. Multiplex immunofluorescence staining was performed using tyramide signal amplification (TSA). Primary antibodies included RAD18 (18333-1-AP, Proteintech), CD68 (97778, CST), CD86 (19589, CST), CD206 (GB113497, Servicebio), CD163 (16646-1-AP, Proteintech), CD8 (66868-1-Ig, Proteintech) and PD-L1 (66248-1-Ig, Proteintech). Horseradish peroxidase‑conjugated secondary antibodies (SeraCare) and TSA kits (FITC‑TSA, CY3‑TSA, CY5‑TSA; Wuhan Baiqiandu Biotechnology) were used for signal detection. After each round of staining, followed by antibody stripping. Nuclei were counterstained with DAPI in the final step. Multiplex images were analyzed using Caseviewer (CV 2.3, CV 2.0) and Pannoramic viewer (PV1.15.3) software, with positive cells quantified at the single‑cell level.

### Immunohistochemistry

The FFPE sections were deparaffinized with xylene and rehydrated using a graded ethanol series. After antigen retrieval, endogenous peroxidase activity was blocked with hydrogen peroxide, and 5% bovine serum albumin (BSA) was applied to reduce non‑specific binding. Sections were then incubated overnight at 4 °C with primary antibodies against RAD18 (18333‑1‑AP; 1:300; Proteintech), CD8 (clone 4SM15; 1:500; eBioscience), or GZMB (ab255598; 1:200; Abcam). This was followed by incubation with horseradish peroxidase‑conjugated secondary antibody at 37 °C for 30 min. Visualization was performed using DAB chromogen for 3–5 min, and sections were counterstained with hematoxylin, dehydrated, cleared, and mounted. For quantitative analysis, five random high‑power fields were captured per sample, and the number of positive cells and staining intensity were scored by two independent observers blinded to the group information.

### Statistical analysis

Bioinformatics analysis was carried out by means of R software (version 4.2.1) and online platforms. Experimental results were analyzed and visualized with GraphPad Prism software (version 8.0.1). Student’s *t* test, one-way/two-way ANOVA (with Tukey’s multiple comparison test for post-hoc analysis), Spearman test, and log-rank test were used to perform intergroup difference analysis, correlation analysis, and prognostic analysis, respectively. Statistical significance was indicated when *p* < 0.05.

## Supplementary information

RAD18-Supplementary files.

## Data Availability

The datasets analyzed in this study are available online from the TCGA, GTEx, and GEO databases, as detailed in the Methods section. All other relevant data can be obtained from the corresponding author upon reasonable request.No new algorithms were developed for this article. All code generated for analysis is available from the authors upon request.
