# Supplementary material for: RAD18 facilitates cancer progression and immunosuppression via the AKT/mTOR/c-Myc axis: a multi-omics analysis
Source: NPJ Precis Oncol. 2026 May 11;10:282. doi: 10.1038/s41698-026-01468-0 (PMC13385966; doi:10.1038/s41698-026-01468-0)

Supplementary Material

**1 Supplementary Figures**


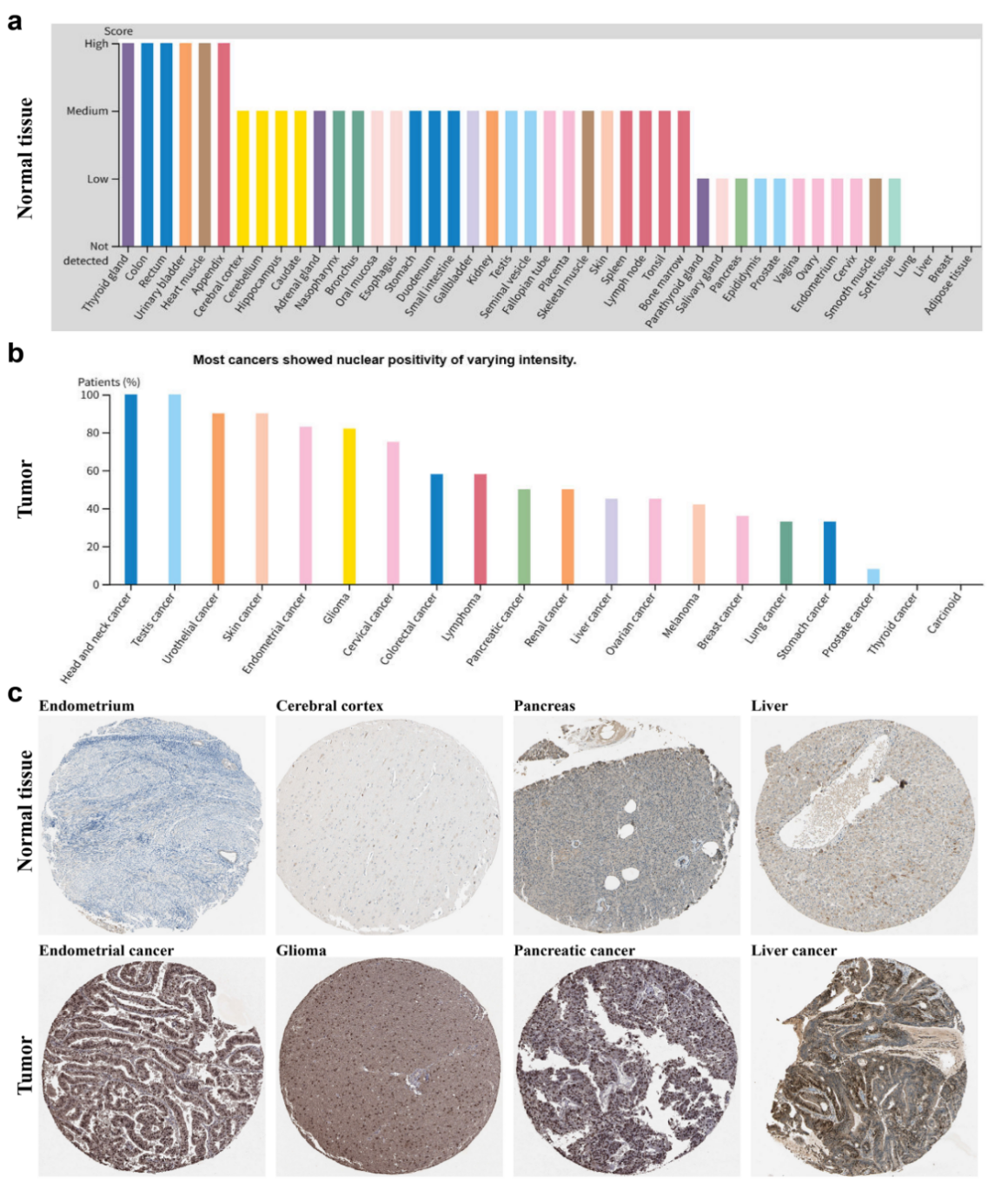


**Supplementary Fig. 1 Expression pattern and staining characteristics of RAD18 in normal and tumor tissues.**

**a** Distribution of RAD18 protein expression levels in normal tissues, with different colors representing expression levels (High: high expression; Medium: moderate expression; Low: low expression; Not detected: not detected), derived from the Human Protein Atlas. **b** The staining positivity rate of RAD18 protein in various tumor types, shown as the percentage of positive cases in each tumor type, derived from the Human Protein Atlas. **c** Comparison of RAD18 protein immunohistochemical staining between normal tissues (upper row: endometrium, cerebral cortex, pancreas, liver) and corresponding tumor tissues (lower row: endometrial carcinoma, glioma, pancreatic cancer, hepatocellular carcinoma), displaying differences in staining intensity, localization, and pattern, derived from the Human Protein Atlas.


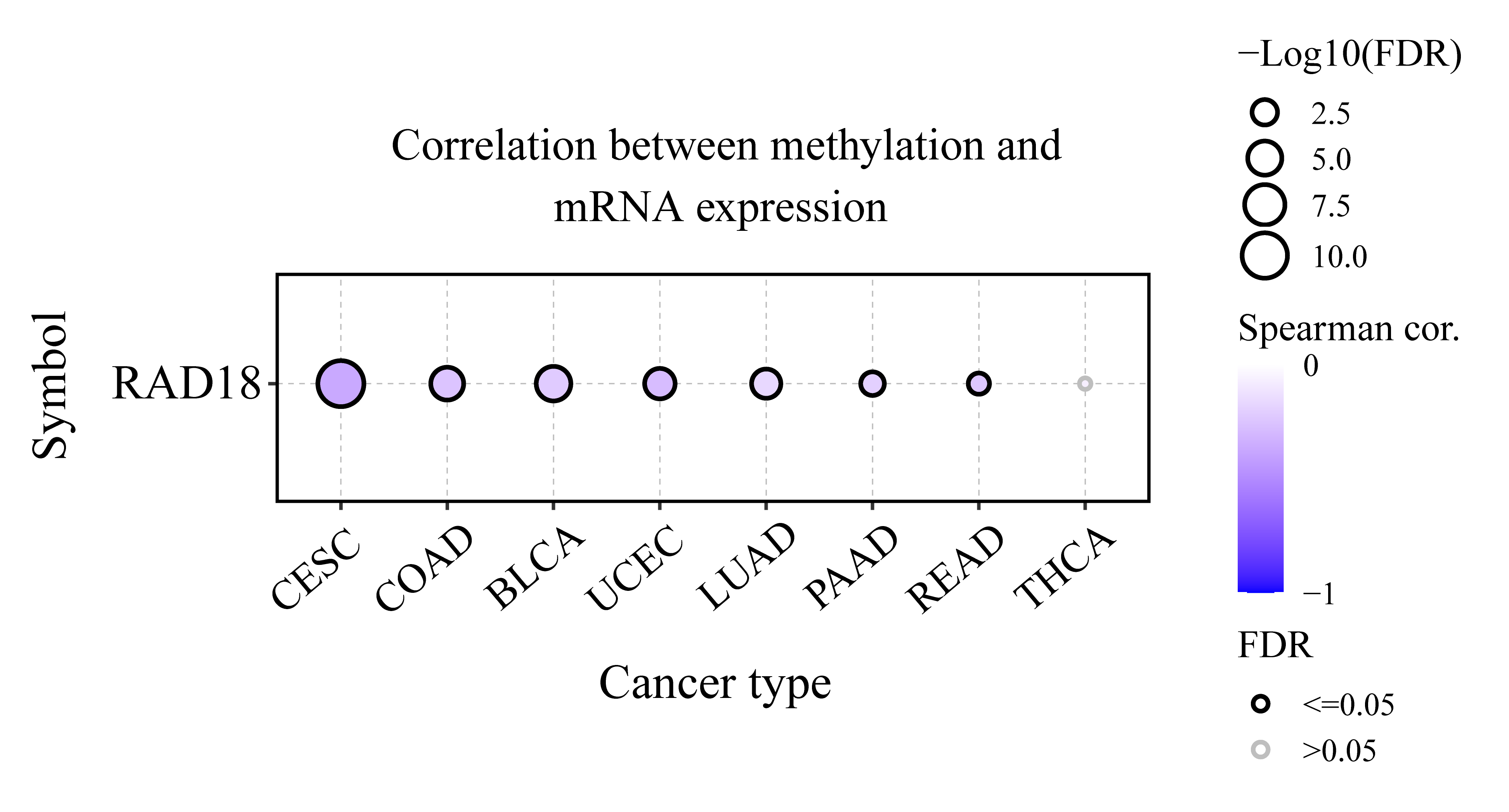


**Supplementary Fig. 2 Correlation between methylation and RAD18 mRNA expression.**

CESC, cervical squamous cell carcinoma and endocervical adenocarcinoma; COAD, colon adenocarcinoma; BLCA, bladder urothelial carcinoma; UCEC, uterine corpus endometrial carcinoma; LUAD, lung adenocarcinoma; PAAD, pancreatic adenocarcinoma; READ, rectum adenocarcinoma; THCA, thyroid carcinoma.


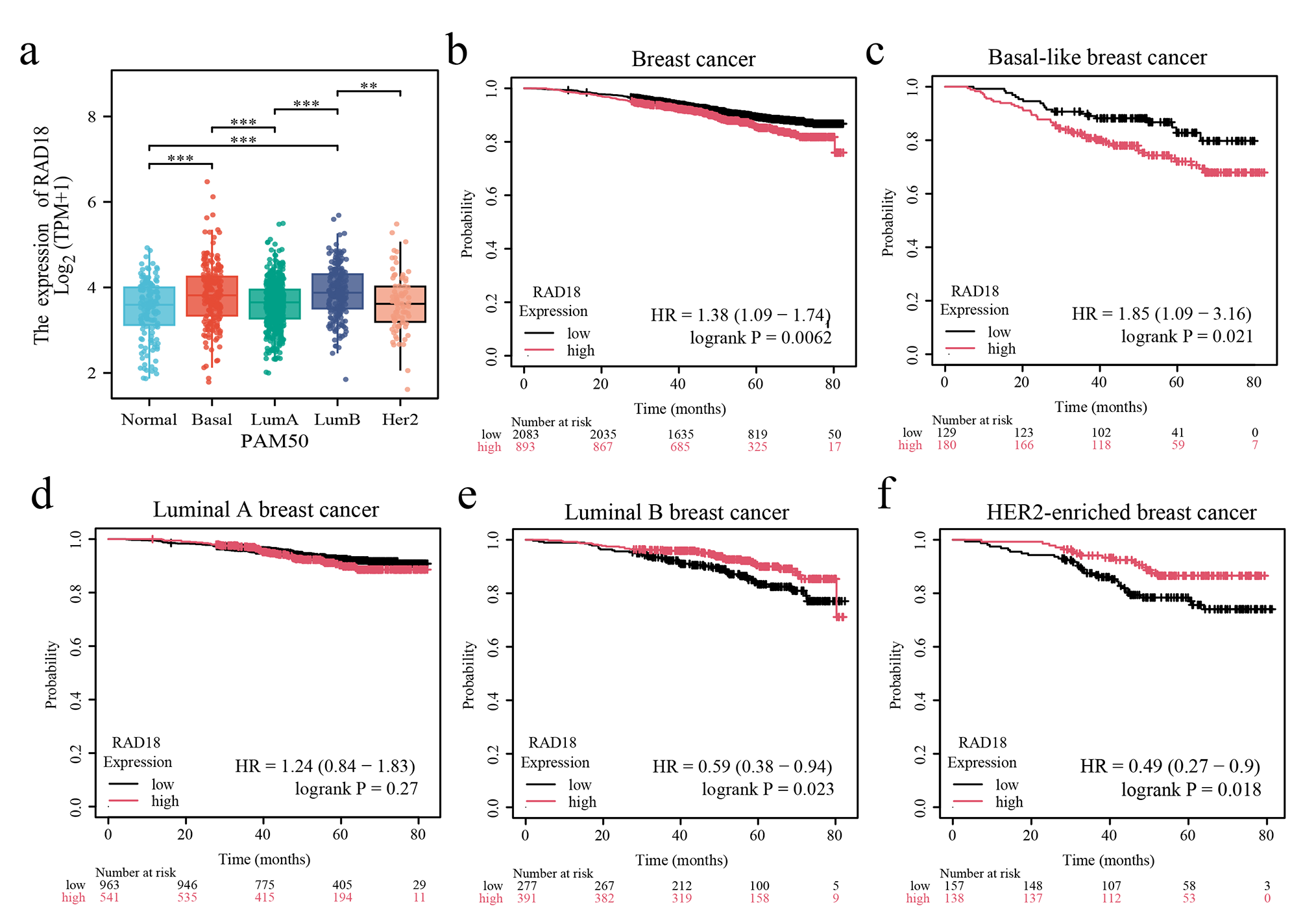


**Supplementary Fig. 3 RAD18 expression and survival outcomes in breast cancer.**

**a** RAD18 expression in normal breast and PAM50 subtypes (****p* < 0.001, ***p* < 0.01). **b–f** Kaplan–Meier survival curves for all breast cancer (b), Basal-like (c), Luminal A (d), Luminal B (e), and HER2-enriched (f) subtypes, stratified by RAD18 expression (low/high). HR: hazard ratio; TPM: transcripts per million.


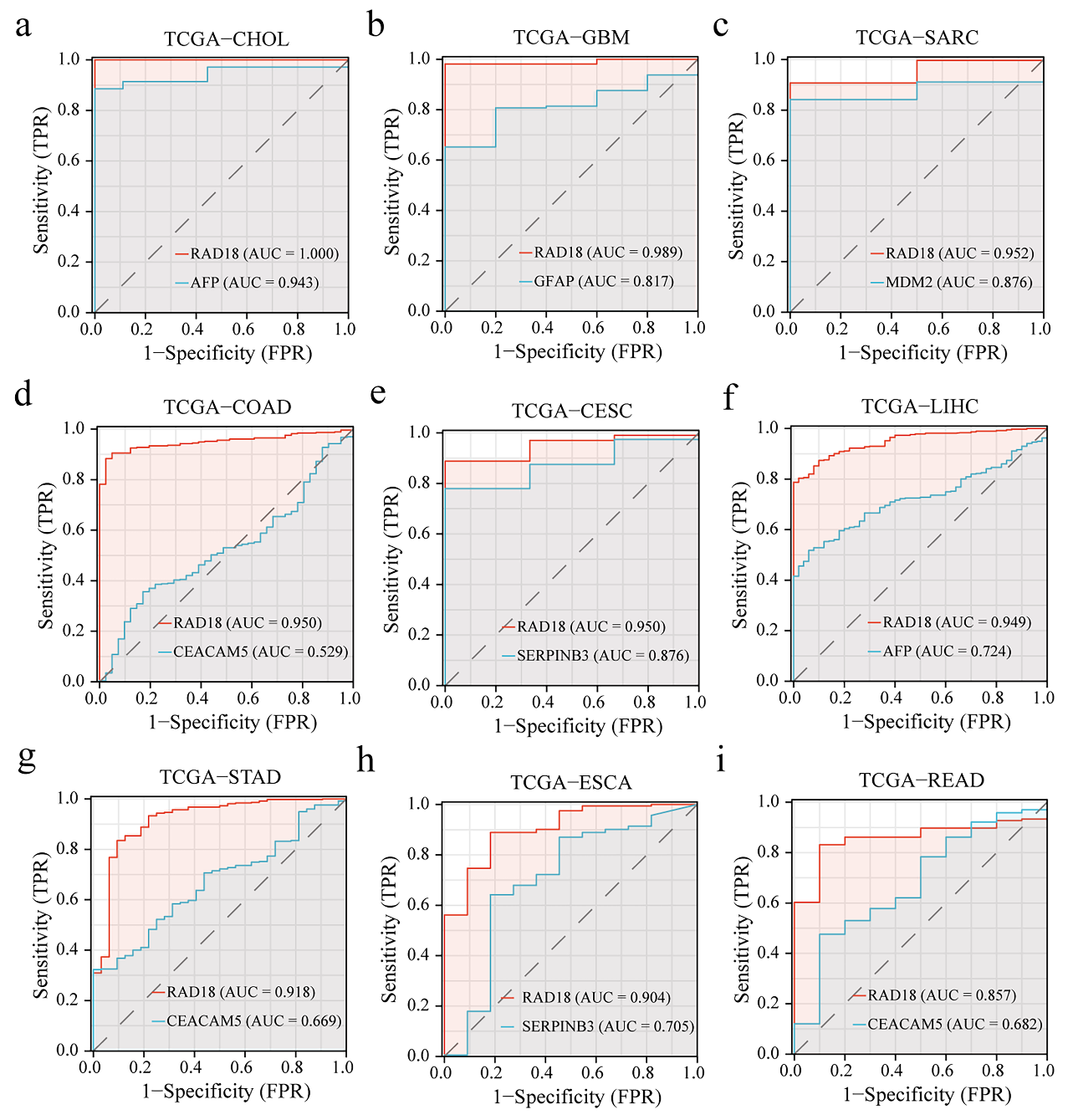


**Supplementary Fig. 4 The area under the receiver operating characteristic (ROC) curve (AUC) was used to compare the diagnostic performance of RAD18 with traditional markers in various cancers.**

**a-i** Based on the AUC values, the diagnostic performance of RAD18 for CHOL (a), GBM (b), SARC (c), COAD (d), CESC (e), LIHC (f), STAD (g), ESCA (h), and READ (i) was higher than that of traditional markers. CHOL, cholangiocarcinoma; GBM, glioblastoma multiforme; SARC,sarcoma; COAD, colon adenocarcinoma; CESC, cervical squamous cell carcinoma and endocervical adenocarcinoma; LIHC, liver hepatocellular carcinoma; STAD, stomach adenocarcinoma; ESCA, esophageal carcinoma; READ, rectum adenocarcinoma.


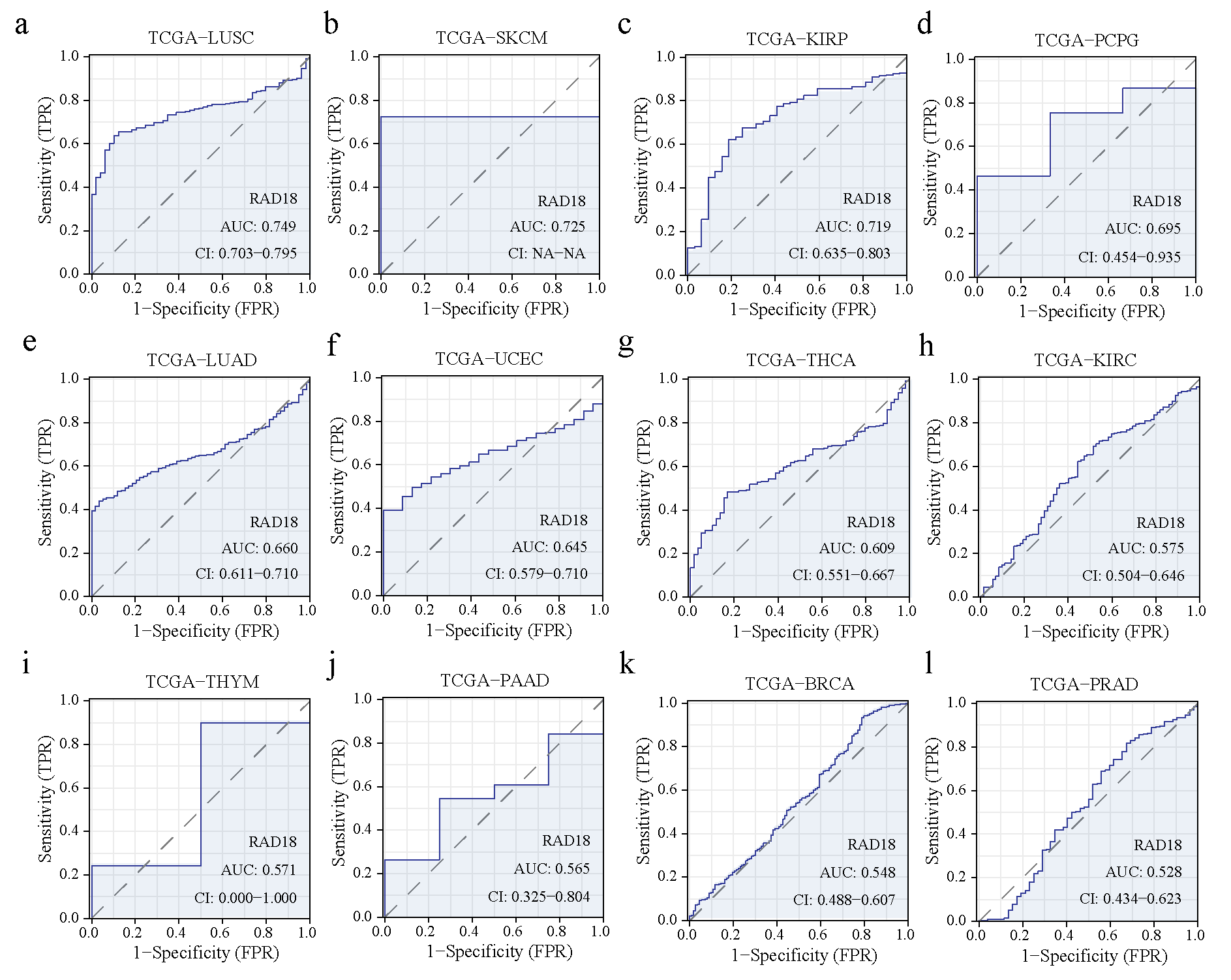


**Supplementary Fig. 5 The diagnostic performance of various cancers was assessed using the areas under the receiver operating characteristic (ROC) curve (AUC), with the AUC values being less than 0.75.**

**a-l** AUC values of RAD18 in LUSC (a), SKCM (b), KIRP (c), PCPG (d), LUAD (e), UCEC (f), THCA (g), KIRC (h), THYM (i), PAAD (j), BRCA (k) and PRAD (l). LUSC, lung squamous cell carcinoma; SKCM, skin cutaneous melanoma; KIRP, kidney renal papillary cell carcinoma; PCPG, pheochromocytoma and paraganglioma; LUAD, lung adenocarcinoma; UCEC, uterine corpus endometrial carcinoma; THCA, thyroid carcinoma; KIRC, kidney renal clear cell carcinoma; THYM, thymoma; PAAD, pancreatic adenocarcinoma; BRCA, breast invasive carcinoma; PRAD, prostate adenocarcinoma.


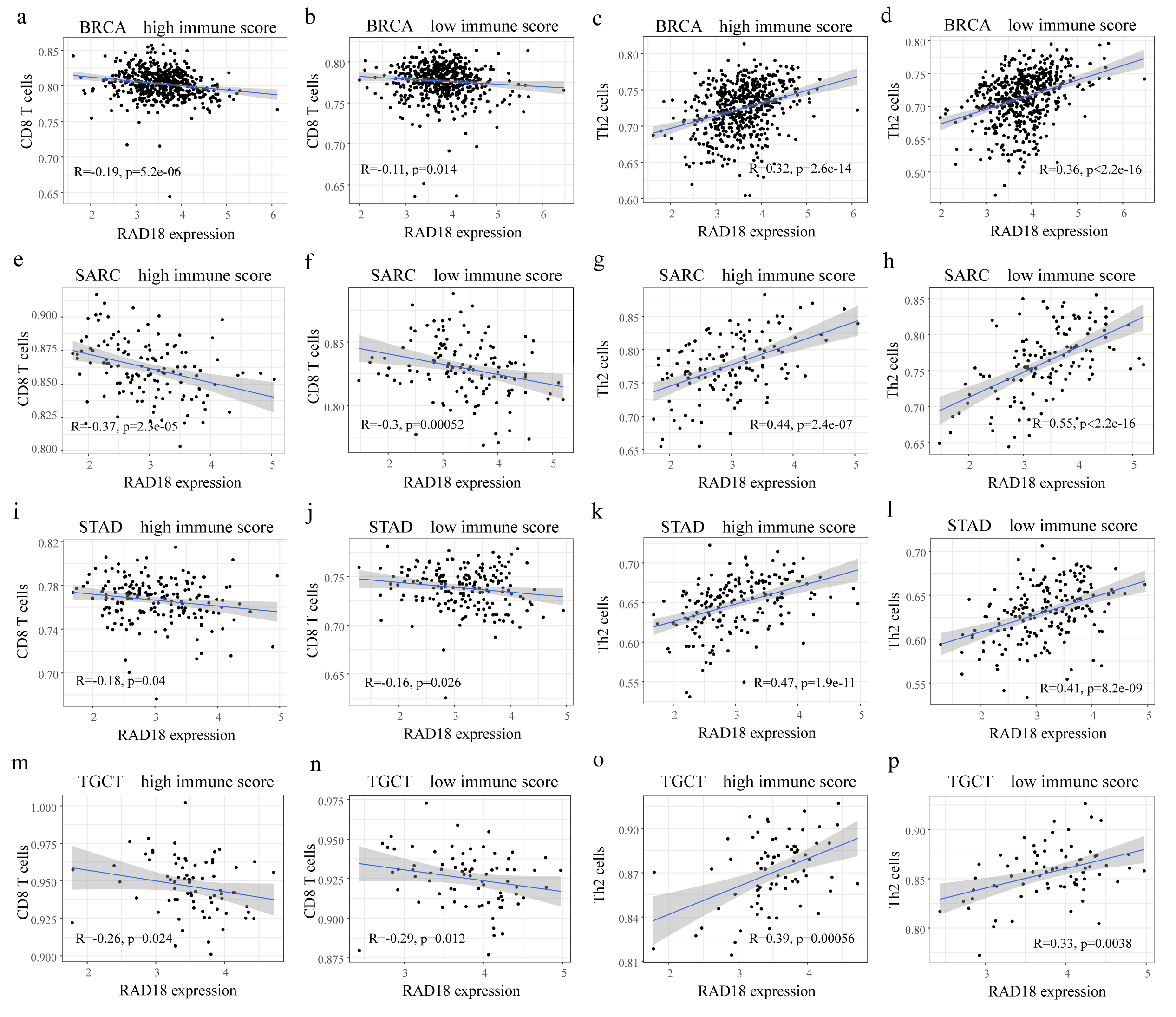


**Supplementary Fig. 6 Correlation between RAD18 expression and immune cell infiltration in BRCA, SARC, STAD, and TGCT cohorts.**

**a-p** The results showed that RAD18 expression was significantly negatively correlated with CD8⁺ T cell infiltration in all four cancer cohorts, regardless of whether the immune score was high or low (all *p* < 0.05, a, b, e, f, i, j, m, n). Furthermore, RAD18 expression was significantly positively correlated with Th2 cell infiltration (all *p* < 0.01, c, d, g, h, k, l, o, p). BRCA, breast invasive carcinoma; SARC,sarcoma; STAD, stomach adenocarcinoma; TGCT, testicular germ cell tumor.


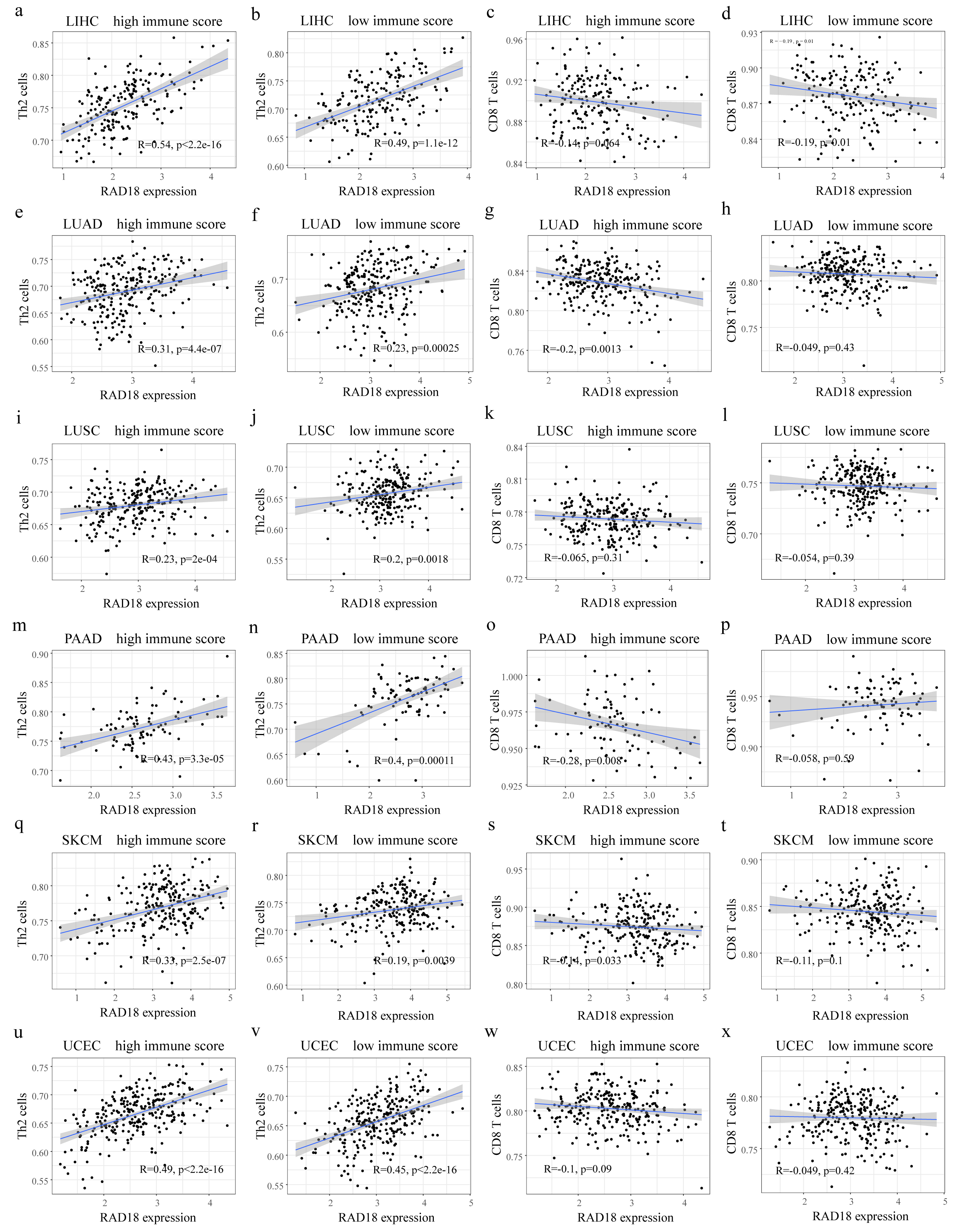


**Supplementary Fig. 7 Correlation between RAD18 expression and immune cell infiltration in LIHC, LUAD, LUSC, PAAD, SKCM, UCEC cohorts.**

**a-x** Results show that RAD18 expression was significantly positively correlated with Th2 cell infiltration in all six cancer cohorts, regardless of whether the immune score was high or low (all *p* < 0.01, a, b, e, f, i, j, m, n, q, r, u, v). However, a significant negative correlation between RAD18 expression and CD8⁺T cell infiltration was not observed in either subgroup or in both subgroups (c, d, g, h, k, l, o, p, s, t, w, x). LIHC, liver hepatocellular carcinoma; LUAD, lung adenocarcinoma; LUSC, lung squamous cell carcinoma; PAAD, pancreatic adenocarcinoma; SKCM, skin cutaneous melanoma; UCEC, uterine corpus endometrial carcinoma.


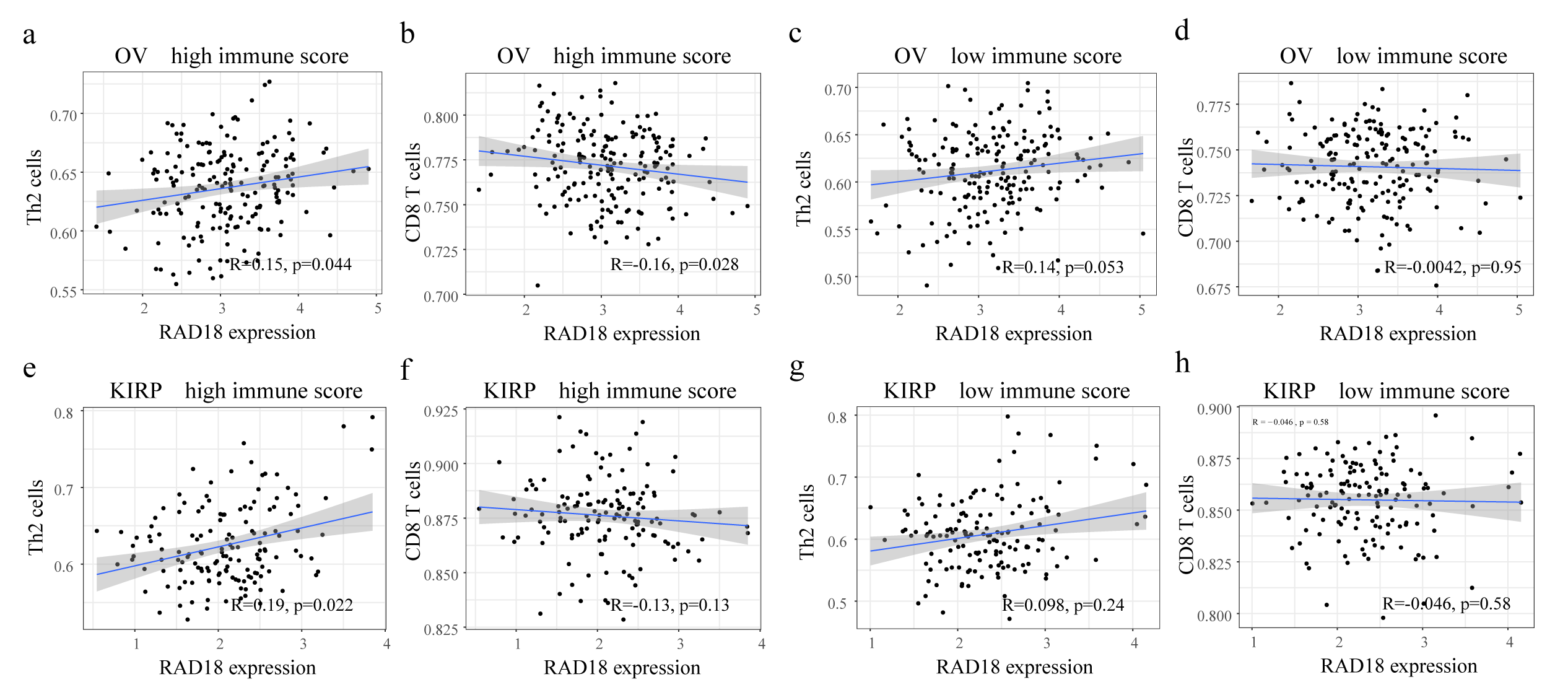


**Supplementary Fig. 8 Correlation between RAD18 expression and immune cell infiltration in OV and KIRP cohorts.**

**a-d** In the OV cohort, RAD18 expression was significantly positively correlated with Th2 cell infiltration and negatively correlated with CD8⁺T cell infiltration only in the high immune score group, but not in the low immune score group. **e-h** In the KIRP cohort, RAD18 expression was significantly positively correlated with Th2 cell infiltration only in the high immune score group, but not in the other groups. OV, ovarian serous cystadenocarcinoma; KIRP, kidney renal papillary cell carcinoma.


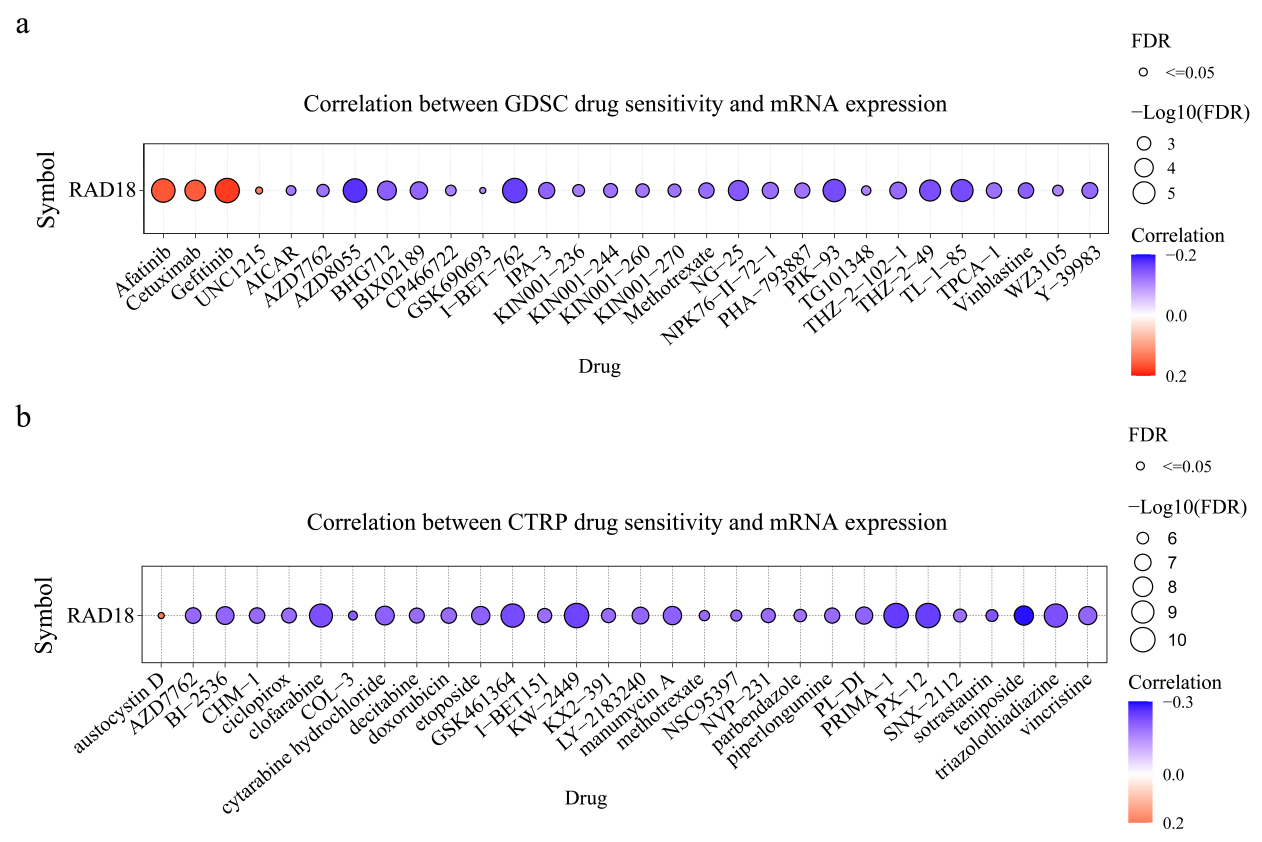


**Supplementary Fig. 9 RAD18 expression and small molecule drug sensitivity.**

**a, b** The correlation between RAD18 expression and the sensitivity to anticancer drugs was analyzed using the GDSC (a) and CTRP (b) datasets.

**
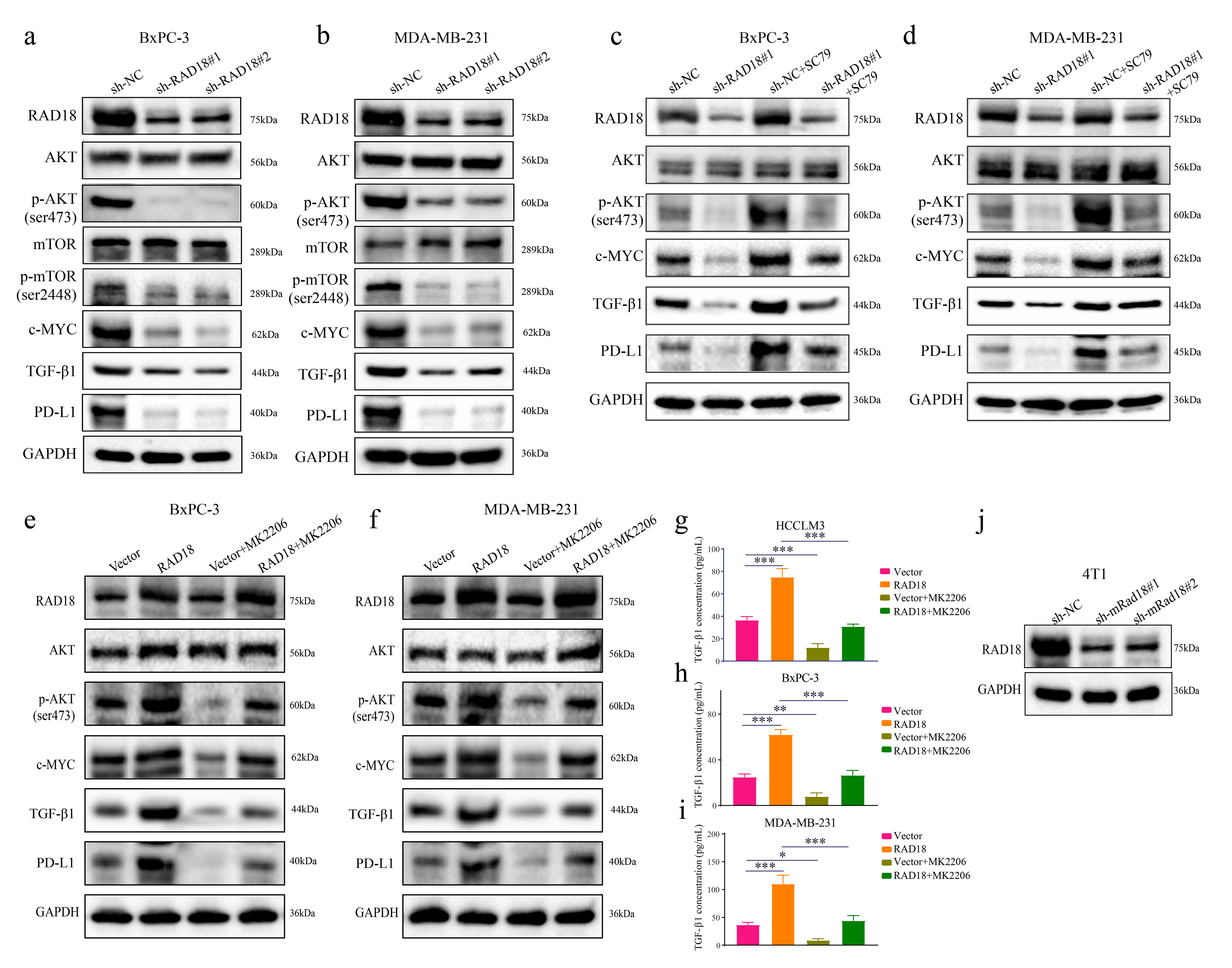
**

**Supplementary Fig. 10 RAD18 activates AKT/mTOR/c-MYC signaling to upregulate TGF-β1 and PD-L1 in cancer cells.**

**a, b** Western blot analysis of RAD18, total AKT, phosphorylated AKT (Ser473), total mTOR, phosphorylated mTOR (Ser2448), c-MYC, TGF-β1, PD-L1, and GAPDH in BxPC-3 (a) and MDA-MB-231 (b) cells transfected with sh-NC or two independent sh-RAD18 constructs (sh-RAD18#1, sh-RAD18#2). **c, d** Western blot analysis of the indicated proteins in BxPC-3 (c) and MDA-MB-231 (d) cells treated with sh-NC, sh-RAD18#1, or sh-RAD18#1 plus SC79 (AKT activator). **e, f** Western blot analysis of the indicated proteins in BxPC-3 (e) and MDA-MB-231 (f) cells transfected with Vector or RAD18, with or without MK2206 (AKT inhibitor) treatment. **g–i** ELISA measurement of TGF-β1 concentration in the culture supernatant of HCCLM3 (g), BxPC-3 (h), and MDA-MB-231 (i) cells under the indicated treatments (Vector, RAD18, Vector+MK2206, RAD18+MK2206). **j** Western blot analysis of RAD18 and GAPDH in 4T1 cells transfected with sh-NC or two independent sh-mRad18 constructs (sh-mRad18#1, sh-mRad18#2). GAPDH was used as a loading control for all Western blot analyses.

**2 Supplementary Tables**

**Supplementary Table1. The full names and abbreviations of each tumor type involved in this study**

| **Abbreviations** | **Full name** |
| --- | --- |
| ACC | Adrenocortical Cancer |
| BLCA | Bladder Urothelial Carcinoma |
| BRCA | Breast invasive carcinoma |
| CESC | Cervical squamous cell carcinoma and endocervical adenocarcinoma |
| CHOL | Cholangiocarcinoma |
| COAD | Colon adenocarcinoma |
| DLBC | Lymphoid Neoplasm Diffuse Large B-cell Lymphoma |
| ESCA | Esophageal carcinoma |
| GBM | Glioblastoma multiforme |
| GBMLGG | Glioma |
| HNSC | Head and Neck squamous cell carcinoma |
| KICH | Kidney Chromophobe |
| KIRC | Kidney renal clear cell carcinoma |
| KIRP | Kidney renal papillary cell carcinoma |
| LAML | Acute Myeloid Leukemia |
| LGG | Brain Lower Grade Glioma |
| LIHC | Liver hepatocellular carcinoma |
| LUAD | Lung adenocarcinoma |
| LUSC | Lung squamous cell carcinoma |
| MESO | Mesothelioma |
| OV | Ovarian serous cystadenocarcinoma |
| PAAD | Pancreatic adenocarcinoma |
| PCPG | Pheochromocytoma and Paraganglioma |
| PRAD | Prostate adenocarcinoma |
| READ | Rectum adenocarcinoma |
| SARC | Sarcoma |
| SKCM | Skin Cutaneous Melanoma |
| STAD | Stomach adenocarcinoma |
| TGCT | Testicular Germ Cell Tumors |
| THCA | Thyroid carcinoma |
| THYM | Thymoma |
| UCEC | Uterine Corpus Endometrial Carcinoma |
| UCS | Uterine Carcinosarcoma |
| UVM | Uveal Melanoma |

**Supplementary Table 2. The average occurrence frequency and proportion of five genetic changes in 32 types of cancer.**

| **Cancer cohorts** | **Alteration Frequency (%)** | **Alteration Type** | **Alteration Count** | **Patient Count** | **Average Alteration Count** | **Average Alteration Frequency (%)** |
| --- | --- | --- | --- | --- | --- | --- |
| BLCA | 7.30 | Amplification | 30 | 411 | **2.81** | **0.82** |
| UCEC | 0.19 | Amplification | 1 | 529 |  |  |
| SARC | 3.14 | Amplification | 8 | 255 |  |  |
| UCS | 1.75 | Amplification | 1 | 57 |  |  |
| OV | 2.05 | Amplification | 12 | 584 |  |  |
| STAD | 0.91 | Amplification | 4 | 440 |  |  |
| SKCM | 0.45 | Amplification | 2 | 442 |  |  |
| ESCA | 1.10 | Amplification | 2 | 182 |  |  |
| KIRC | 0.00 | Amplification | 0 | 512 |  |  |
| THYM | 0.00 | Amplification | 0 | 123 |  |  |
| COAD | 0.00 | Amplification | 0 | 594 |  |  |
| MESO | 1.15 | Amplification | 1 | 87 |  |  |
| LUSC | 0.00 | Amplification | 0 | 487 |  |  |
| BRCA | 1.48 | Amplification | 16 | 1084 |  |  |
| PRAD | 0.40 | Amplification | 2 | 494 |  |  |
| CESC | 0.34 | Amplification | 1 | 297 |  |  |
| HNSC | 0.19 | Amplification | 1 | 523 |  |  |
| LUAD | 0.18 | Amplification | 1 | 566 |  |  |
| LGG | 0.78 | Amplification | 4 | 514 |  |  |
| PCPG | 0.56 | Amplification | 1 | 178 |  |  |
| GBM | 0.34 | Amplification | 2 | 585 |  |  |
| KIRP | 0.00 | Amplification | 0 | 283 |  |  |
| LIHC | 0.27 | Amplification | 1 | 372 |  |  |
| LAML | 0.00 | Amplification | 0 | 200 |  |  |
| ACC | 0.00 | Amplification | 0 | 92 |  |  |
| CHOL | 0.00 | Amplification | 0 | 36 |  |  |
| DLBC | 0.00 | Amplification | 0 | 48 |  |  |
| KICH | 0.00 | Amplification | 0 | 65 |  |  |
| PAAD | 0.00 | Amplification | 0 | 184 |  |  |
| TGCT | 0.00 | Amplification | 0 | 149 |  |  |
| THCA | 0.00 | Amplification | 0 | 500 |  |  |
| UVM | 0.00 | Amplification | 0 | 80 |  |  |
| BLCA | 0.49 | Mutation | 2 | 411 | **2.91** | **0.85** |
| UCEC | 4.91 | Mutation | 26 | 529 |  |  |
| SARC | 0.78 | Mutation | 2 | 255 |  |  |
| UCS | 1.75 | Mutation | 1 | 57 |  |  |
| OV | 0.34 | Mutation | 2 | 584 |  |  |
| STAD | 1.36 | Mutation | 6 | 440 |  |  |
| SKCM | 2.26 | Mutation | 10 | 442 |  |  |
| ESCA | 0.55 | Mutation | 1 | 182 |  |  |
| KIRC | 0.00 | Mutation | 0 | 512 |  |  |
| THYM | 0.81 | Mutation | 1 | 123 |  |  |
| COAD | 2.36 | Mutation | 14 | 594 |  |  |
| MESO | 1.15 | Mutation | 1 | 87 |  |  |
| LUSC | 1.23 | Mutation | 6 | 487 |  |  |
| BRCA | 0.55 | Mutation | 6 | 1084 |  |  |
| PRAD | 0.61 | Mutation | 3 | 494 |  |  |
| CESC | 0.67 | Mutation | 2 | 297 |  |  |
| HNSC | 0.76 | Mutation | 4 | 523 |  |  |
| LUAD | 0.71 | Mutation | 4 | 566 |  |  |
| LGG | 0.00 | Mutation | 0 | 514 |  |  |
| PCPG | 0.00 | Mutation | 0 | 178 |  |  |
| GBM | 0.17 | Mutation | 1 | 585 |  |  |
| KIRP | 0.35 | Mutation | 1 | 283 |  |  |
| LIHC | 0.00 | Mutation | 0 | 372 |  |  |
| LAML | 0.00 | Mutation | 0 | 200 |  |  |
| ACC | 0.00 | Mutation | 0 | 92 |  |  |
| CHOL | 0.00 | Mutation | 0 | 36 |  |  |
| DLBC | 0.00 | Mutation | 0 | 48 |  |  |
| KICH | 0.00 | Mutation | 0 | 65 |  |  |
| PAAD | 0.00 | Mutation | 0 | 184 |  |  |
| TGCT | 0.00 | Mutation | 0 | 149 |  |  |
| THCA | 0.00 | Mutation | 0 | 500 |  |  |
| UVM | 0.00 | Mutation | 0 | 80 |  |  |
| BLCA | 0.00 | Deep Deletion | 0 | 411 | **1.16** | **0.34** |
| UCEC | 0.00 | Deep Deletion | 0 | 529 |  |  |
| SARC | 0.39 | Deep Deletion | 1 | 255 |  |  |
| UCS | 0.00 | Deep Deletion | 0 | 57 |  |  |
| OV | 0.51 | Deep Deletion | 3 | 584 |  |  |
| STAD | 0.45 | Deep Deletion | 2 | 440 |  |  |
| SKCM | 0.23 | Deep Deletion | 1 | 442 |  |  |
| ESCA | 1.10 | Deep Deletion | 2 | 182 |  |  |
| KIRC | 2.54 | Deep Deletion | 13 | 512 |  |  |
| THYM | 1.63 | Deep Deletion | 2 | 123 |  |  |
| COAD | 0.00 | Deep Deletion | 0 | 594 |  |  |
| MESO | 0.00 | Deep Deletion | 0 | 87 |  |  |
| LUSC | 1.03 | Deep Deletion | 5 | 487 |  |  |
| BRCA | 0.00 | Deep Deletion | 0 | 1084 |  |  |
| PRAD | 1.01 | Deep Deletion | 5 | 494 |  |  |
| CESC | 0.67 | Deep Deletion | 2 | 297 |  |  |
| HNSC | 0.00 | Deep Deletion | 0 | 523 |  |  |
| LUAD | 0.18 | Deep Deletion | 1 | 566 |  |  |
| LGG | 0.00 | Deep Deletion | 0 | 514 |  |  |
| PCPG | 0.00 | Deep Deletion | 0 | 178 |  |  |
| GBM | 0.00 | Deep Deletion | 0 | 585 |  |  |
| KIRP | 0.00 | Deep Deletion | 0 | 283 |  |  |
| LIHC | 0.00 | Deep Deletion | 0 | 372 |  |  |
| LAML | 0.00 | Deep Deletion | 0 | 200 |  |  |
| ACC | 0.00 | Deep Deletion | 0 | 92 |  |  |
| CHOL | 0.00 | Deep Deletion | 0 | 36 |  |  |
| DLBC | 0.00 | Deep Deletion | 0 | 48 |  |  |
| KICH | 0.00 | Deep Deletion | 0 | 65 |  |  |
| PAAD | 0.00 | Deep Deletion | 0 | 184 |  |  |
| TGCT | 0.00 | Deep Deletion | 0 | 149 |  |  |
| THCA | 0.00 | Deep Deletion | 0 | 500 |  |  |
| UVM | 0.00 | Deep Deletion | 0 | 80 |  |  |
| BLCA | 0.24 | Multiple Alterations | 1 | 411 | **0.06** | **0.02** |
| UCEC | 0.00 | Multiple Alterations | 0 | 529 |  |  |
| SARC | 0.00 | Multiple Alterations | 0 | 255 |  |  |
| UCS | 0.00 | Multiple Alterations | 0 | 57 |  |  |
| OV | 0.00 | Multiple Alterations | 0 | 584 |  |  |
| STAD | 0.23 | Multiple Alterations | 1 | 440 |  |  |
| SKCM | 0.00 | Multiple Alterations | 0 | 442 |  |  |
| ESCA | 0.00 | Multiple Alterations | 0 | 182 |  |  |
| KIRC | 0.00 | Multiple Alterations | 0 | 512 |  |  |
| THYM | 0.00 | Multiple Alterations | 0 | 123 |  |  |
| COAD | 0.00 | Multiple Alterations | 0 | 594 |  |  |
| MESO | 0.00 | Multiple Alterations | 0 | 87 |  |  |
| LUSC | 0.00 | Multiple Alterations | 0 | 487 |  |  |
| BRCA | 0.00 | Multiple Alterations | 0 | 1084 |  |  |
| PRAD | 0.00 | Multiple Alterations | 0 | 494 |  |  |
| CESC | 0.00 | Multiple Alterations | 0 | 297 |  |  |
| HNSC | 0.00 | Multiple Alterations | 0 | 523 |  |  |
| LUAD | 0.00 | Multiple Alterations | 0 | 566 |  |  |
| LGG | 0.00 | Multiple Alterations | 0 | 514 |  |  |
| PCPG | 0.00 | Multiple Alterations | 0 | 178 |  |  |
| GBM | 0.00 | Multiple Alterations | 0 | 585 |  |  |
| KIRP | 0.00 | Multiple Alterations | 0 | 283 |  |  |
| LIHC | 0.00 | Multiple Alterations | 0 | 372 |  |  |
| LAML | 0.00 | Multiple Alterations | 0 | 200 |  |  |
| ACC | 0.00 | Multiple Alterations | 0 | 92 |  |  |
| CHOL | 0.00 | Multiple Alterations | 0 | 36 |  |  |
| DLBC | 0.00 | Multiple Alterations | 0 | 48 |  |  |
| KICH | 0.00 | Multiple Alterations | 0 | 65 |  |  |
| PAAD | 0.00 | Multiple Alterations | 0 | 184 |  |  |
| TGCT | 0.00 | Multiple Alterations | 0 | 149 |  |  |
| THCA | 0.00 | Multiple Alterations | 0 | 500 |  |  |
| UVM | 0.00 | Multiple Alterations | 0 | 80 |  |  |
| BLCA | 0.00 | Structural Variant | 0 | 411 | **0.19** | **0.05** |
| UCEC | 0.00 | Structural Variant | 0 | 529 |  |  |
| SARC | 0.00 | Structural Variant | 0 | 255 |  |  |
| UCS | 0.00 | Structural Variant | 0 | 57 |  |  |
| OV | 0.51 | Structural Variant | 3 | 584 |  |  |
| STAD | 0.23 | Structural Variant | 1 | 440 |  |  |
| SKCM | 0.00 | Structural Variant | 0 | 442 |  |  |
| ESCA | 0.00 | Structural Variant | 0 | 182 |  |  |
| KIRC | 0.00 | Structural Variant | 0 | 512 |  |  |
| THYM | 0.00 | Structural Variant | 0 | 123 |  |  |
| COAD | 0.00 | Structural Variant | 0 | 594 |  |  |
| MESO | 0.00 | Structural Variant | 0 | 87 |  |  |
| LUSC | 0.00 | Structural Variant | 0 | 487 |  |  |
| BRCA | 0.09 | Structural Variant | 1 | 1084 |  |  |
| PRAD | 0.00 | Structural Variant | 0 | 494 |  |  |
| CESC | 0.00 | Structural Variant | 0 | 297 |  |  |
| HNSC | 0.19 | Structural Variant | 1 | 523 |  |  |
| LUAD | 0.00 | Structural Variant | 0 | 566 |  |  |
| LGG | 0.00 | Structural Variant | 0 | 514 |  |  |
| PCPG | 0.00 | Structural Variant | 0 | 178 |  |  |
| GBM | 0.00 | Structural Variant | 0 | 585 |  |  |
| KIRP | 0.00 | Structural Variant | 0 | 283 |  |  |
| LIHC | 0.00 | Structural Variant | 0 | 372 |  |  |
| LAML | 0.00 | Structural Variant | 0 | 200 |  |  |
| ACC | 0.00 | Structural Variant | 0 | 92 |  |  |
| CHOL | 0.00 | Structural Variant | 0 | 36 |  |  |
| DLBC | 0.00 | Structural Variant | 0 | 48 |  |  |
| KICH | 0.00 | Structural Variant | 0 | 65 |  |  |
| PAAD | 0.00 | Structural Variant | 0 | 184 |  |  |
| TGCT | 0.00 | Structural Variant | 0 | 149 |  |  |
| THCA | 0.00 | Structural Variant | 0 | 500 |  |  |
| UVM | 0.00 | Structural Variant | 0 | 80 |  |  |

**Supplementary Table 3. Univariate and multivariate cox regression analyses of RAD18’s independent prognostic role in ACC.**

| Characteristics | Total  (N) | Univariate analysis | |  | Multivariate analysis | |
| --- | --- | --- | --- | --- | --- | --- |
|  |  | Hazard ratio (95% CI) | P value |  | Hazard ratio (95% CI) | P value |
| Age | 77 |  |  |  |  |  |
| <= 50 | 40 | Reference |  |  |  |  |
| > 50 | 37 | 1.929 (0.891 - 4.178) | 0.095 |  |  |  |
| Gender | 77 |  |  |  |  |  |
| Female | 48 | Reference |  |  |  |  |
| Male | 29 | 0.986 (0.451 - 2.154) | 0.972 |  |  |  |
| Pathologic T stage | 77 |  |  |  |  |  |
| T1&T2 | 51 | Reference |  |  | Reference |  |
| T3&T4 | 26 | 10.286 (3.976 - 26.608) | **< 0.001** |  | 10.037 (1.164 - 86.559) | **0.036** |
| Pathologic N stage | 77 |  |  |  |  |  |
| N0 | 68 | Reference |  |  |  |  |
| N1 | 9 | 2.038 (0.769 - 5.400) | 0.152 |  |  |  |
| Pathologic M stage | 77 |  |  |  |  |  |
| M0 | 62 | Reference |  |  | Reference |  |
| M1 | 15 | 6.150 (2.710 - 13.959) | **< 0.001** |  | 1.141 (0.416 - 3.123) | 0.798 |
| Pathologic stage | 77 |  |  |  |  |  |
| Stage I&Stage II | 46 | Reference |  |  | Reference |  |
| Stage III&Stage IV | 31 | 6.476 (2.706 - 15.498) | **< 0.001** |  | 0.646 (0.078 - 5.332) | 0.685 |
| RAD18 | 77 |  |  |  |  |  |
| Low-RAD18 expressers | 38 | Reference |  |  | Reference |  |
| High-RAD18 expressers | 39 | 5.909 (2.350 - 14.860) | **< 0.001** |  | 4.011 (1.454 - 11.070) | **0.007** |

ACC, adrenocortical carcinoma

**Supplementary Table 4. Univariate and multivariate cox regression analyses of RAD18’s independent prognostic role in MESO.**

| Characteristics | Total  (N) | Univariate analysis | |  | Multivariate analysis | |
| --- | --- | --- | --- | --- | --- | --- |
|  |  | Hazard ratio (95% CI) | P value |  | Hazard ratio (95% CI) | P value |
| Age | 57 |  |  |  |  |  |
| <= 65 | 27 | Reference |  |  |  |  |
| > 65 | 30 | 1.592 (0.900 - 2.816) | 0.110 |  |  |  |
| Gender | 57 |  |  |  |  |  |
| Female | 12 | Reference |  |  |  |  |
| Male | 45 | 0.710 (0.363 - 1.390) | 0.318 |  |  |  |
| Pathologic T stage | 57 |  |  |  |  |  |
| T1&T2 | 28 | Reference |  |  |  |  |
| T3&T4 | 29 | 0.853 (0.488 - 1.491) | 0.577 |  |  |  |
| Pathologic N stage | 57 |  |  |  |  |  |
| N0&N1 | 38 | Reference |  |  |  |  |
| N2&N3 | 19 | 0.632 (0.343 - 1.165) | 0.141 |  |  |  |
| Pathologic M stage | 57 |  |  |  |  |  |
| M0 | 54 | Reference |  |  |  |  |
| M1 | 3 | 1.830 (0.434 - 7.720) | 0.410 |  |  |  |
| Pathologic stage | 57 |  |  |  |  |  |
| Stage I&Stage II | 18 | Reference |  |  |  |  |
| Stage III&Stage IV | 39 | 0.855 (0.466 - 1.568) | 0.613 |  |  |  |
| RAD18 | 57 |  |  |  |  |  |
| Low-RAD18 expressers | 27 | Reference |  |  | Reference |  |
| High-RAD18 expressers | 30 | 2.568 (1.420 - 4.642) | **0.002** |  | 2.568 (1.420 - 4.642) | **0.002** |

MESO, mesothelioma

**Supplementary Table 5. Univariate and multivariate cox regression analyses of RAD18’s independent prognostic role in LGG.**

| Characteristics | Total  (N) | Univariate analysis | |  | Multivariate analysis | |
| --- | --- | --- | --- | --- | --- | --- |
|  |  | Hazard ratio (95% CI) | P value |  | Hazard ratio (95% CI) | P value |
| Age | 466 |  |  |  |  |  |
| <= 40 | 231 | Reference |  |  | Reference |  |
| > 40 | 235 | 2.735 (1.843 - 4.058) | **< 0.001** |  | 2.817 (1.840 - 4.313) | **< 0.001** |
| Gender | 466 |  |  |  |  |  |
| Female | 207 | Reference |  |  |  |  |
| Male | 259 | 1.141 (0.788 - 1.652) | 0.486 |  |  |  |
| WHO grade | 466 |  |  |  |  |  |
| G2 | 221 | Reference |  |  | Reference |  |
| G3 | 245 | 3.033 (2.021 - 4.552) | **< 0.001** |  | 1.780 (1.135 - 2.791) | **0.012** |
| IDH status | 466 |  |  |  |  |  |
| Wild | 89 | Reference |  |  | Reference |  |
| Mutant | 377 | 0.186 (0.126 - 0.272) | **< 0.001** |  | 0.307 (0.194 - 0.485) | **< 0.001** |
| 1p/19q codeletion | 466 |  |  |  |  |  |
| Non-codel | 313 | Reference |  |  | Reference |  |
| Codel | 153 | 0.416 (0.261 - 0.664) | **< 0.001** |  | 0.606 (0.360 - 1.022) | 0.061 |
| RAD18 | 466 |  |  |  |  |  |
| Low-RAD18 expressers | 232 | Reference |  |  | Reference |  |
| High-RAD18 expressers | 234 | 2.844 (1.897 - 4.263) | **< 0.001** |  | 1.885 (1.228 - 2.893) | **0.004** |

LGG, brain lower grade glioma; WHO, world health organization; IDH, isocitrate dehydrogenase

**Supplementary Table 6. Univariate and multivariate cox regression analyses of RAD18’s independent prognostic role in LIHC.**

| Characteristics | Total  (N) | Univariate analysis | |  | Multivariate analysis | |
| --- | --- | --- | --- | --- | --- | --- |
|  |  | Hazard ratio (95% CI) | P value |  | Hazard ratio (95% CI) | P value |
| Age | 238 |  |  |  |  |  |
| <= 60 | 131 | Reference |  |  |  |  |
| > 60 | 107 | 1.205 (0.767 - 1.894) | 0.418 |  |  |  |
| Gender | 238 |  |  |  |  |  |
| Female | 75 | Reference |  |  |  |  |
| Male | 163 | 0.751 (0.472 - 1.194) | 0.226 |  |  |  |
| Pathologic T stage | 238 |  |  |  |  |  |
| T1&T2 | 170 | Reference |  |  | Reference |  |
| T3&T4 | 68 | 3.125 (1.986 - 4.919) | **< 0.001** |  | 1.963 (0.267 - 14.437) | 0.508 |
| Pathologic N stage | 238 |  |  |  |  |  |
| N0 | 234 | Reference |  |  |  |  |
| N1 | 4 | 2.111 (0.516 - 8.641) | 0.299 |  |  |  |
| Pathologic M stage | 238 |  |  |  |  |  |
| M0 | 234 | Reference |  |  |  |  |
| M1 | 4 | 3.996 (1.253 - 12.750) | **0.019** |  |  |  |
| Pathologic stage | 238 |  |  |  |  |  |
| Stage I&Stage II | 166 | Reference |  |  | Reference |  |
| Stage III&Stage IV | 72 | 3.107 (1.975 - 4.889) | **< 0.001** |  | 1.464 (0.198 - 10.813) | 0.709 |
| RAD18 | 238 |  |  |  |  |  |
| Low-RAD18 expressers | 106 | Reference |  |  | Reference |  |
| High-RAD18 expressers | 132 | 2.052 (1.264 - 3.332) | **0.004** |  | 1.726 (1.053 - 2.828) | **0.030** |

LIHC, liver hepatocellular carcinoma

**Supplementary Table 7. Univariate and multivariate cox regression analyses of RAD18’s independent prognostic role in PAAD.**

| Characteristics | Total  (N) | Univariate analysis | |  | Multivariate analysis | |
| --- | --- | --- | --- | --- | --- | --- |
|  |  | Hazard ratio (95% CI) | P value |  | Hazard ratio (95% CI) | P value |
| Age | 179 |  |  |  |  |  |
| <= 65 | 94 | Reference |  |  |  |  |
| > 65 | 85 | 1.285 (0.853 - 1.937) | 0.230 |  |  |  |
| Gender | 179 |  |  |  |  |  |
| Female | 80 | Reference |  |  |  |  |
| Male | 99 | 0.813 (0.541 - 1.222) | 0.319 |  |  |  |
| Pathologic T stage | 177 |  |  |  |  |  |
| T1&T2 | 31 | Reference |  |  | Reference |  |
| T3&T4 | 146 | 2.035 (1.079 - 3.838) | **0.028** |  | 1.150 (0.586 - 2.255) | 0.685 |
| Pathologic N stage | 174 |  |  |  |  |  |
| N0 | 50 | Reference |  |  | Reference |  |
| N1 | 124 | 2.161 (1.287 - 3.627) | **0.004** |  | 2.109 (1.215 - 3.664) | **0.008** |
| Pathologic M stage | 85 |  |  |  |  |  |
| M0 | 80 | Reference |  |  |  |  |
| M1 | 5 | 0.773 (0.185 - 3.227) | 0.724 |  |  |  |
| RAD18 | 179 |  |  |  |  |  |
| Low-RAD18 expressers | 89 | Reference |  |  | Reference |  |
| High-RAD18 expressers | 90 | 1.688 (1.113 - 2.559) | **0.014** |  | 1.599 (1.044 - 2.450) | **0.031** |

PAAD, pancreatic adenocarcinoma

**The full uncropped Gels and Blots image(s)**


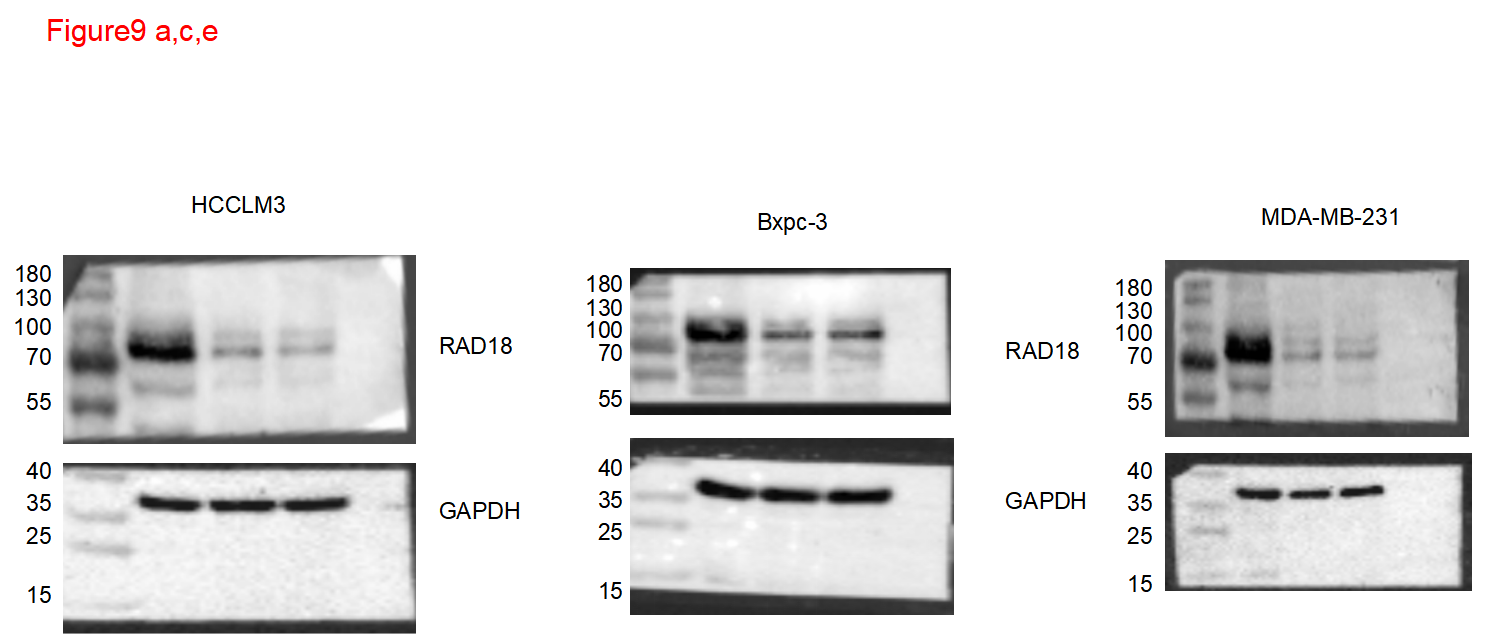

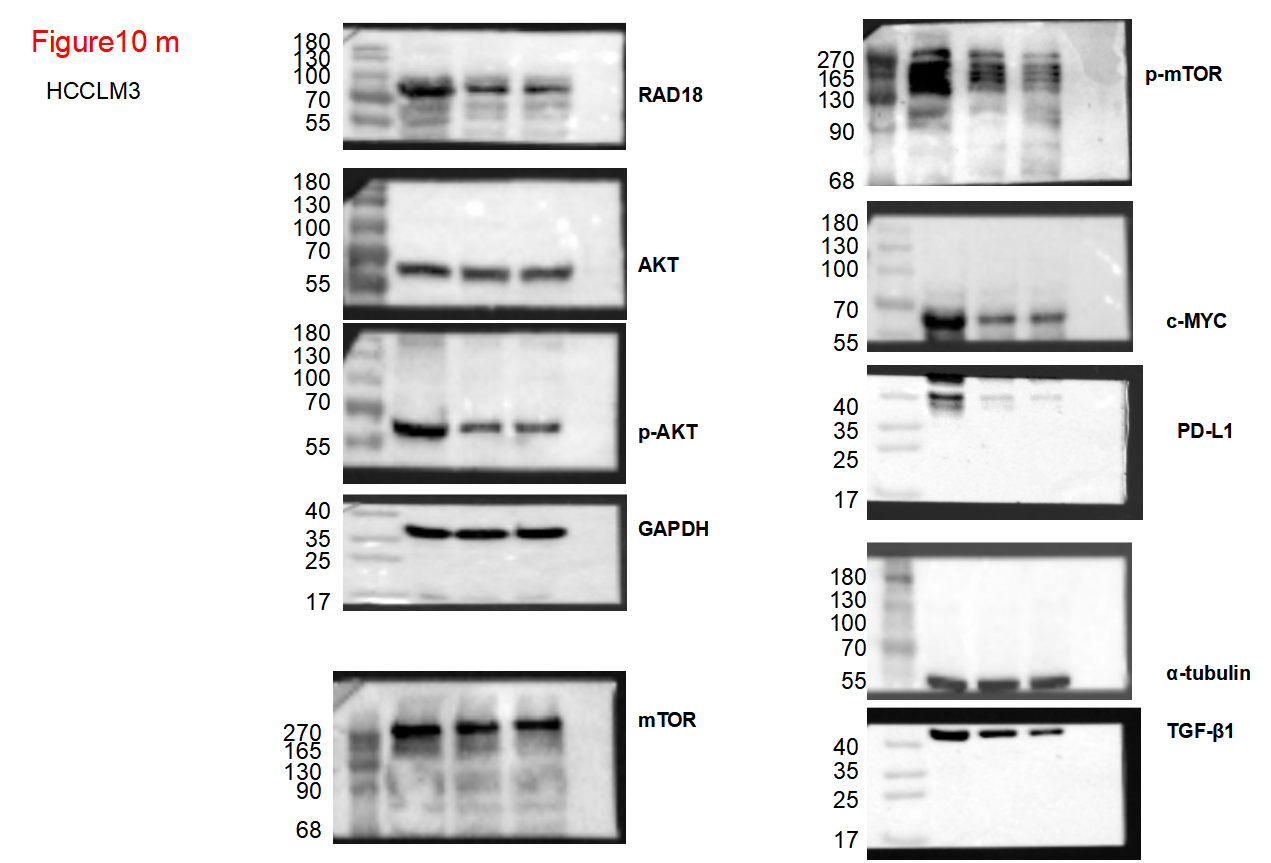

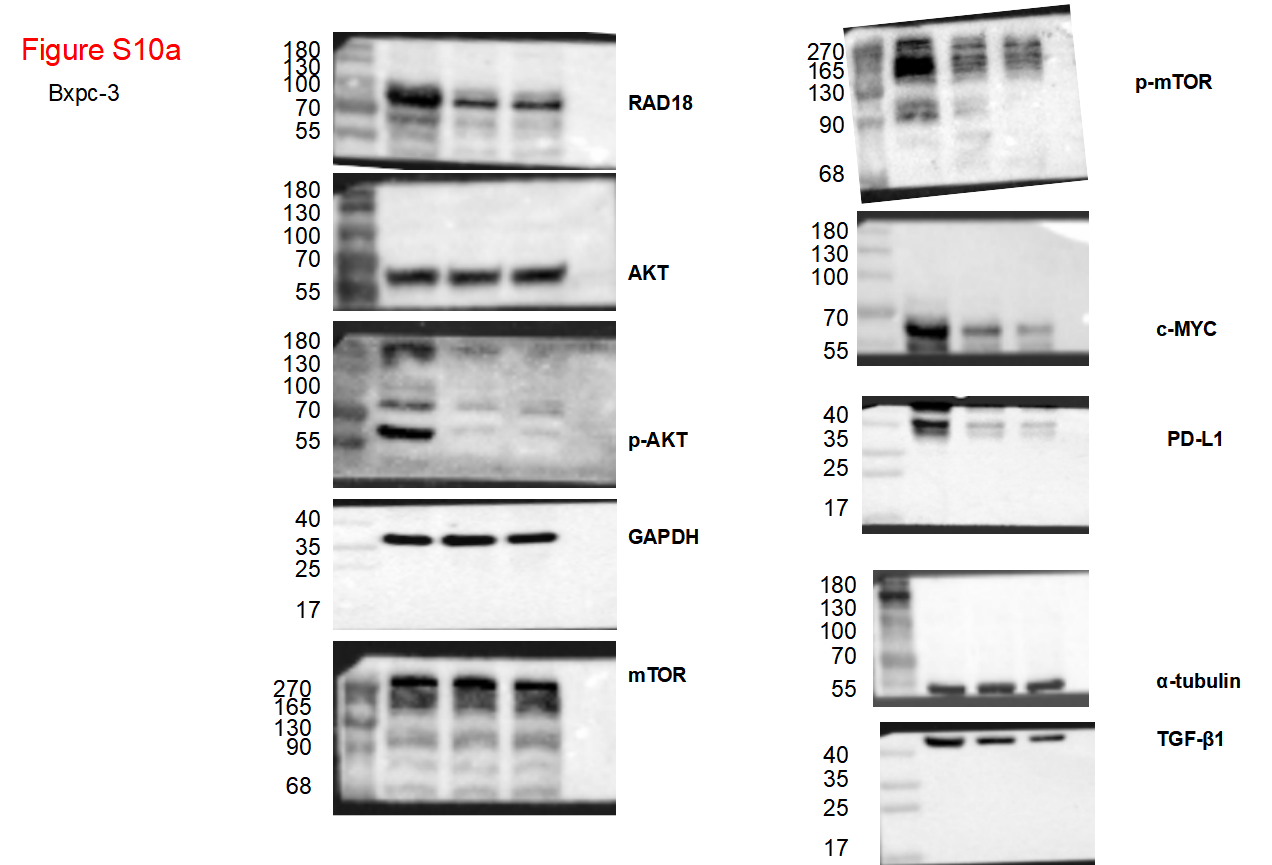

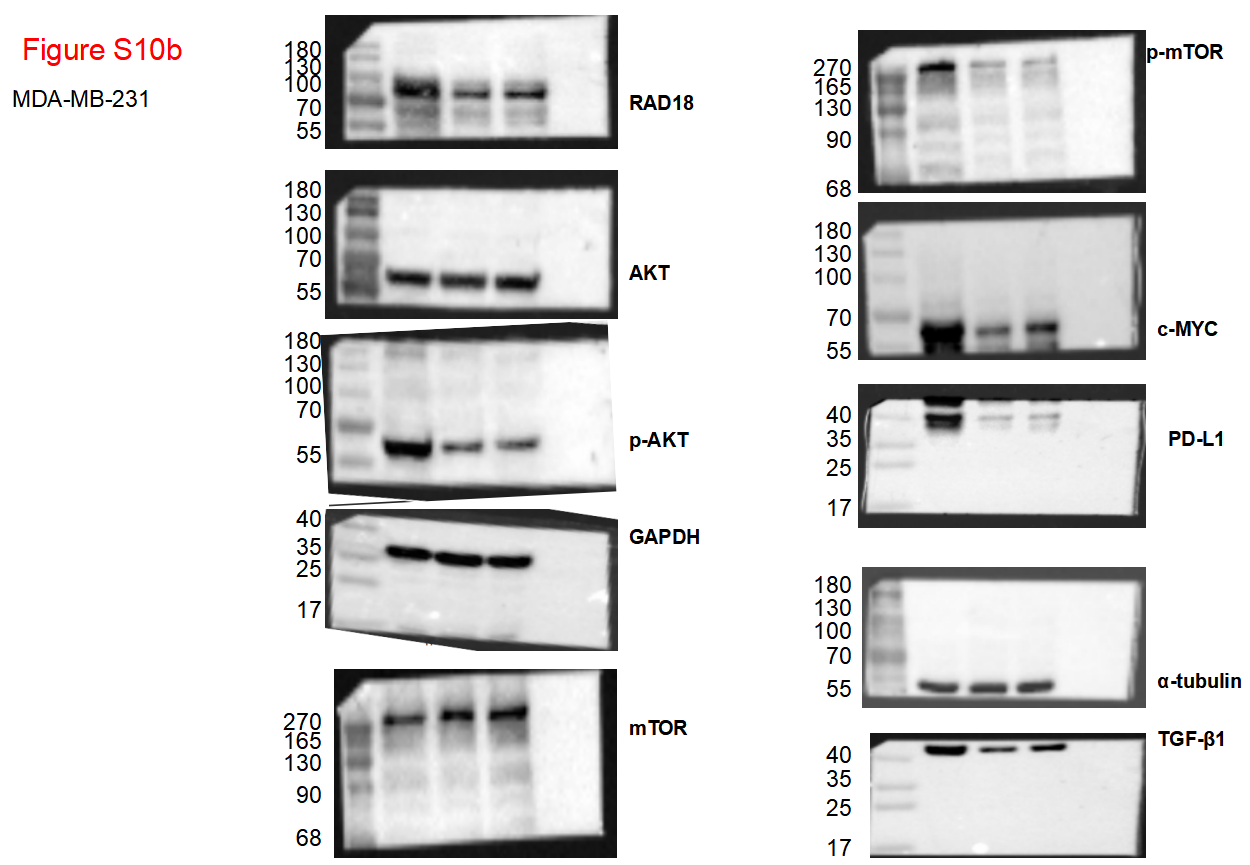

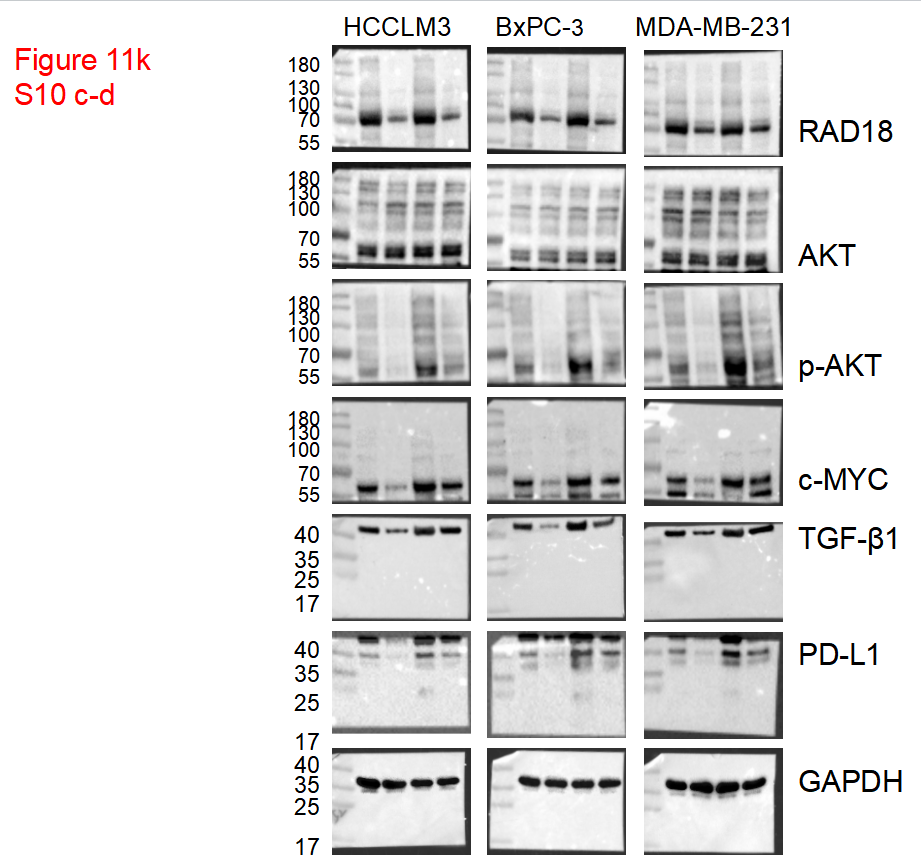

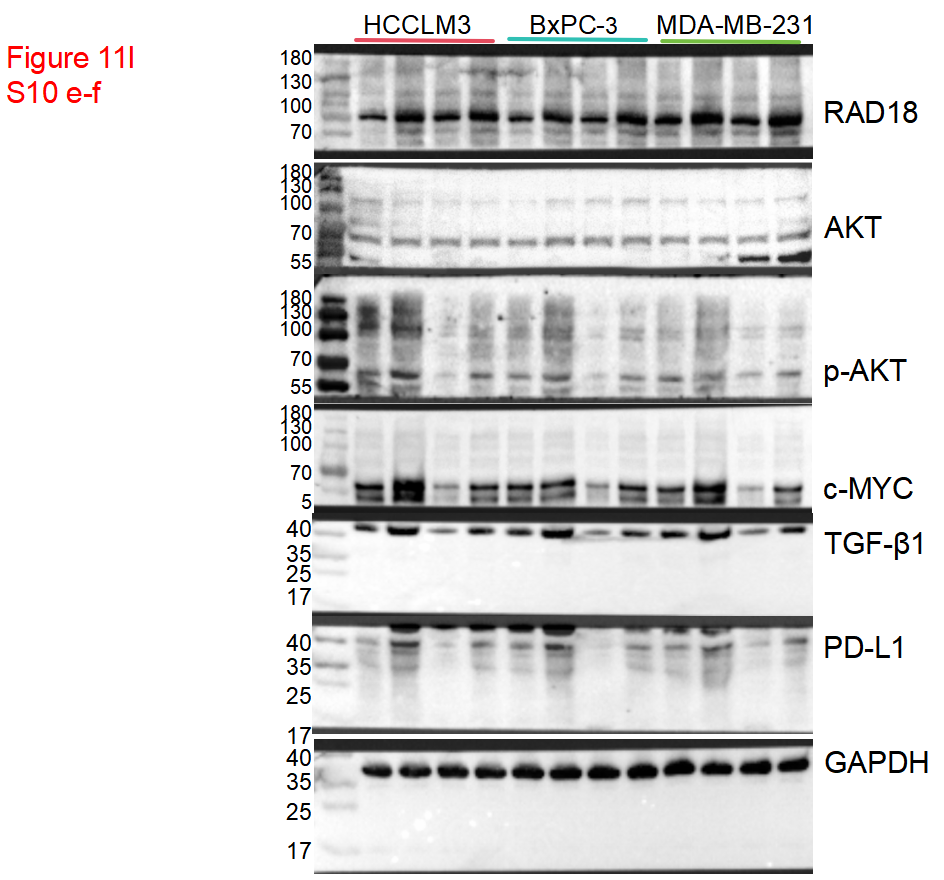

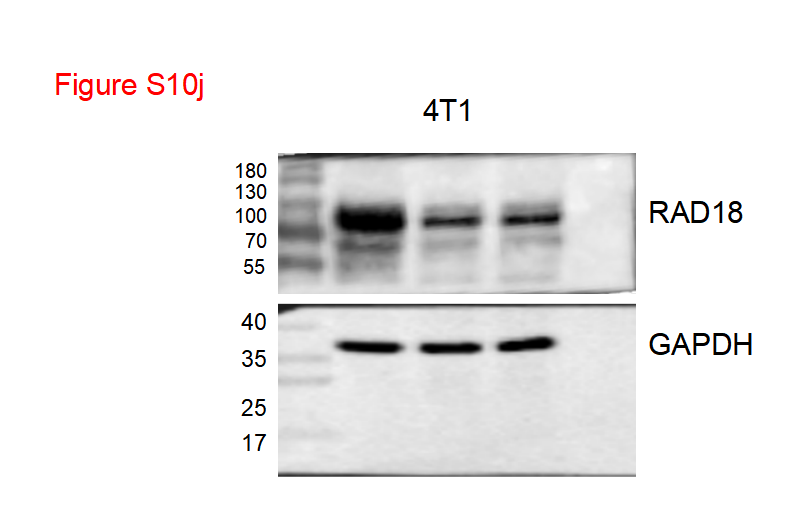

Supplement: Supplementary file 1 — RAD18-Supplementary files. [file 41698_2026_1468_MOESM1_ESM.docx]
